# Discovery and characterization of high-affinity, potent SARS-CoV-2 neutralizing antibodies via single B cell screening

**DOI:** 10.1038/s41598-021-99401-x

**Published:** 2021-10-20

**Authors:** John S. Schardt, Ghasidit Pornnoppadol, Alec A. Desai, Kyung Soo Park, Jennifer M. Zupancic, Emily K. Makowski, Matthew D. Smith, Hongwei Chen, Mayara Garcia de Mattos Barbosa, Marilia Cascalho, Thomas M. Lanigan, James J. Moon, Peter M. Tessier

**Affiliations:** 1 Departments of Chemical Engineering, University of Michigan, Ann Arbor, MI 48109 USA; 2 Pharmaceutical Sciences, University of Michigan, Ann Arbor, MI 48109 USA; 3 Biomedical Engineering, University of Michigan, Ann Arbor, MI 48109 USA; 4 Biointerfaces Institute, University of Michigan, Ann Arbor, MI 48109 USA; 5 Department of Surgery, University of Michigan, Ann Arbor, MI 48109 USA; 6 Department of Microbiology and Immunology, University of Michigan, Ann Arbor, MI 48109 USA; 7 Division of Rheumatology, Department of Internal Medicine, University of Michigan Medical School, Ann Arbor, MI USA; 8 University of Michigan, North Campus Research Complex, B10-179, 2800 Plymouth Road, Ann Arbor, MI 48109 USA

**Keywords:** Antibody generation, Biologics, Applied immunology

## Abstract

Monoclonal antibodies that target SARS-CoV-2 with high affinity are valuable for a wide range of biomedical applications involving novel coronavirus disease (COVID-19) diagnosis, treatment, and prophylactic intervention. Strategies for the rapid and reliable isolation of these antibodies, especially potent neutralizing antibodies, are critical toward improved COVID-19 response and informed future response to emergent infectious diseases. In this study, single B cell screening was used to interrogate antibody repertoires of immunized mice and isolate antigen-specific IgG1^+^ memory B cells. Using these methods, high-affinity, potent neutralizing antibodies were identified that target the receptor-binding domain of SARS-CoV-2. Further engineering of the identified molecules to increase valency resulted in enhanced neutralizing activity. Mechanistic investigation revealed that these antibodies compete with ACE2 for binding to the receptor-binding domain of SARS-CoV-2. These antibodies may warrant further development for urgent COVID-19 applications. Overall, these results highlight the potential of single B cell screening for the rapid and reliable identification of high-affinity, potent neutralizing antibodies for infectious disease applications.

## Introduction

The novel coronavirus disease (COVID-19) pandemic, caused by severe acute respiratory syndrome coronavirus 2 (SARS-CoV-2), has taken a devastating toll on human health and is linked to over one million deaths^1^. To mitigate the burden of COVID-19, widespread access to effective therapeutics, diagnostics, and vaccines is sorely needed. Encouragingly, several vaccine candidates have emerged as timely prophylactic interventions with early indications that are tremendously promising. However, large-scale manufacturing, distribution and administration remain major hurdles, and many people have not yet been vaccinated due to lack of access. Moreover, COVID-19 remains a severe global threat to human health^2^.

Antibodies that bind SARS-CoV-2 with high affinity serve a vital role in combating COVID-19 and complement vaccine-based prophylactic intervention. Indeed, antibodies have been successfully employed for COVID-19 diagnosis, treatment, prophylaxis, and vaccine development^3–7^. Of particular note, several monoclonal antibodies and combinations thereof have received emergency use authorization for the treatment of COVID-19 in patients with mild-to-moderate symptoms with the goal of reducing disease progression and hospitalization^3,4^. These include bamlanivimab and etesevimab from Eli Lilly and casirivimab and imdevimab from Regeneron. Encouragingly, standard antibody discovery and development timelines that include everything from target identification to clinical development—typically ranging from the order of years to decades—have been greatly accelerated, resulting in the rapid discovery, development, and manufacturing of antibodies for urgent COVID-19 applications.

**Figure 1:**
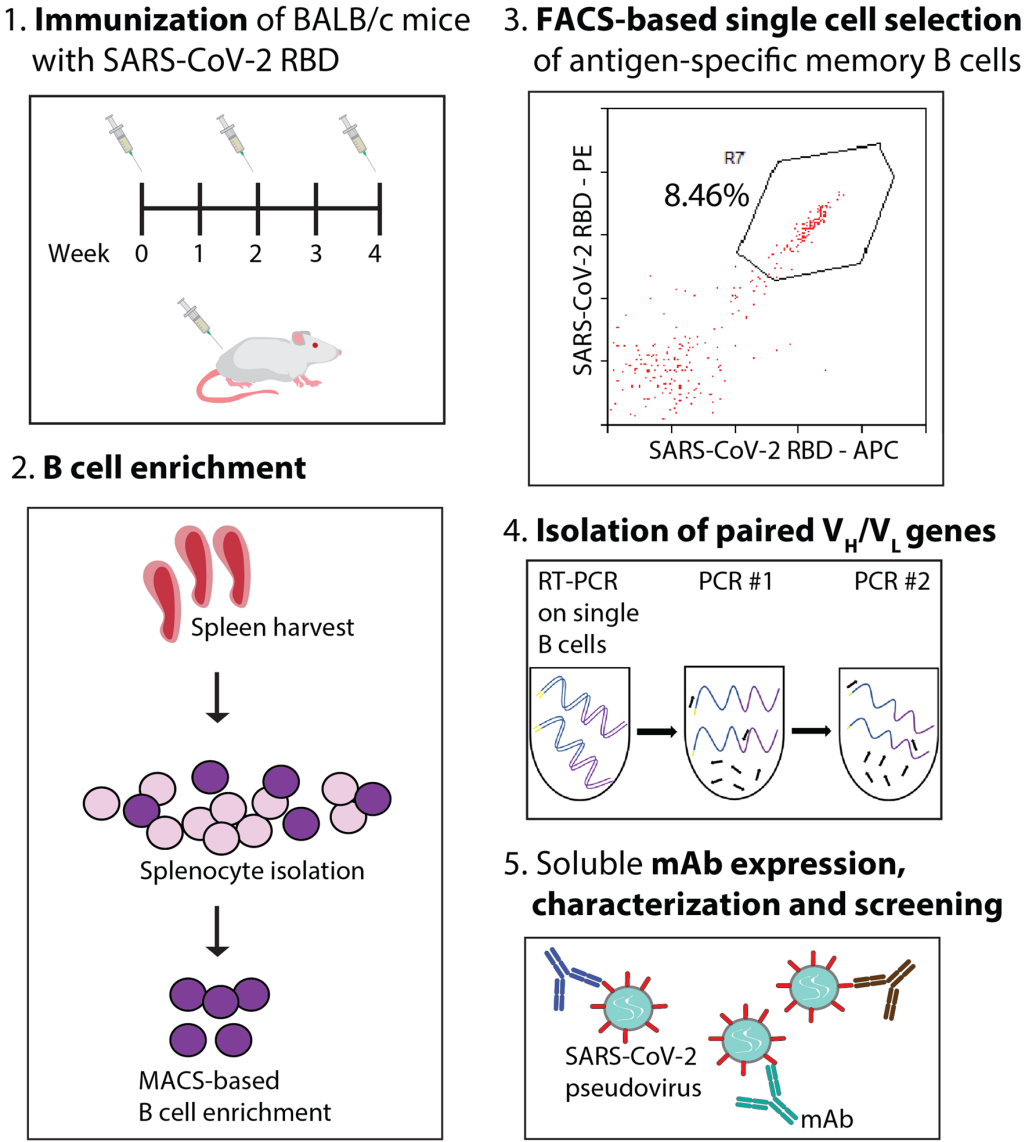
Overview of methodologies for discovery and characterization of potent SARS-CoV-2 neutralizing antibodies isolated from single B cells. (**1**) BALB/c mice were immunized with SARS-CoV-2 RBD three times at 2-week intervals. (**2**) Splenocytes were harvested six weeks following the initial vaccination from which B cells were isolated via MACS and labeled for cell sorting. (**3**) Cells positive for binding to SARS-CoV-2 RBD labeled with two unique fluorophores (gate R7) were sorted as single cells into 96-well plates. (**4**) Multiple PCR steps were employed to isolate V_H_ and V_L_ antibody genes. (**5**) Antibodies were expressed, purified, and evaluated for antigen binding, neutralizing activity, and biophysical properties.

At the early stages of such accelerated development, strategies for the rapid and reliable discovery of high-affinity, potent neutralizing antibodies are critical toward affording timely response to infectious diseases such as COVID-19. Mounting evidence suggests the broad utility of single B cell screening for facile isolation of high-affinity antibodies against a desired target antigen within the order of weeks^8–13^. This process involves the isolation of viable cells (typically sourced from immunized animals or convalescent patients), high-throughput single cell sorting of desired antigen-specific subpopulations, recovery of paired V_H_/V_L_ antibody genes via RT-PCR and PCR steps, and expression and evaluation of antibody candidates. An example of this methodology is illustrated in Fig. 1. Single B cell cloning has resulted in the identification of high-affinity antibodies against a wide variety of antigens using samples from a variety of species (including mouse, rabbit, and human) and tissues (including spleen, bone marrow, and blood)^8–13^. Overall, single B cell screening is attractive given its use of natural antibody repertoires, retention of native V_H_/V_L_ pairing, suitability for high-throughput discovery and evaluation, and simplicity relative to alternative approaches.

The goal of this study was to evaluate the utility of single B cell screening for the isolation of potent neutralizing antibodies against SARS-CoV-2. Notably, previous reports indicate that both high affinity and target epitope are key determinants of antibody neutralizing activity^14,15^ in order to effectively disrupt the high affinity (low nM) interaction of the SARS-CoV-2 receptor-binding domain (RBD) with human ACE2^14–18^. Toward this goal, we sought to identify high-affinity antibodies from IgG1^+^ memory B cells derived from the spleens of immunized mice. For the immunization, a key requirement was to elicit sufficient IgG1^+^ memory B cell response for single cell screening, such that neutralizing antibodies represent a subset of this response. We reasoned that the SARS-CoV-2 RBD combined with the standard adjuvant alum would represent a logical and low-risk vaccine, given that the RBD is implicated in virus infectivity^16^ and alum is linked to strong humoral response^19^. By employing this vaccination approach together with single B cell screening, we report the facile generation, engineering, and characterization of novel antibodies that possess high-affinity and potent neutralizing activity against SARS-CoV-2.

## Results

### Mice immunized with SARS-CoV-2 receptor-binding domain demonstrate significant antigen-specific IgG and IgG1 responses

**Figure 2:**
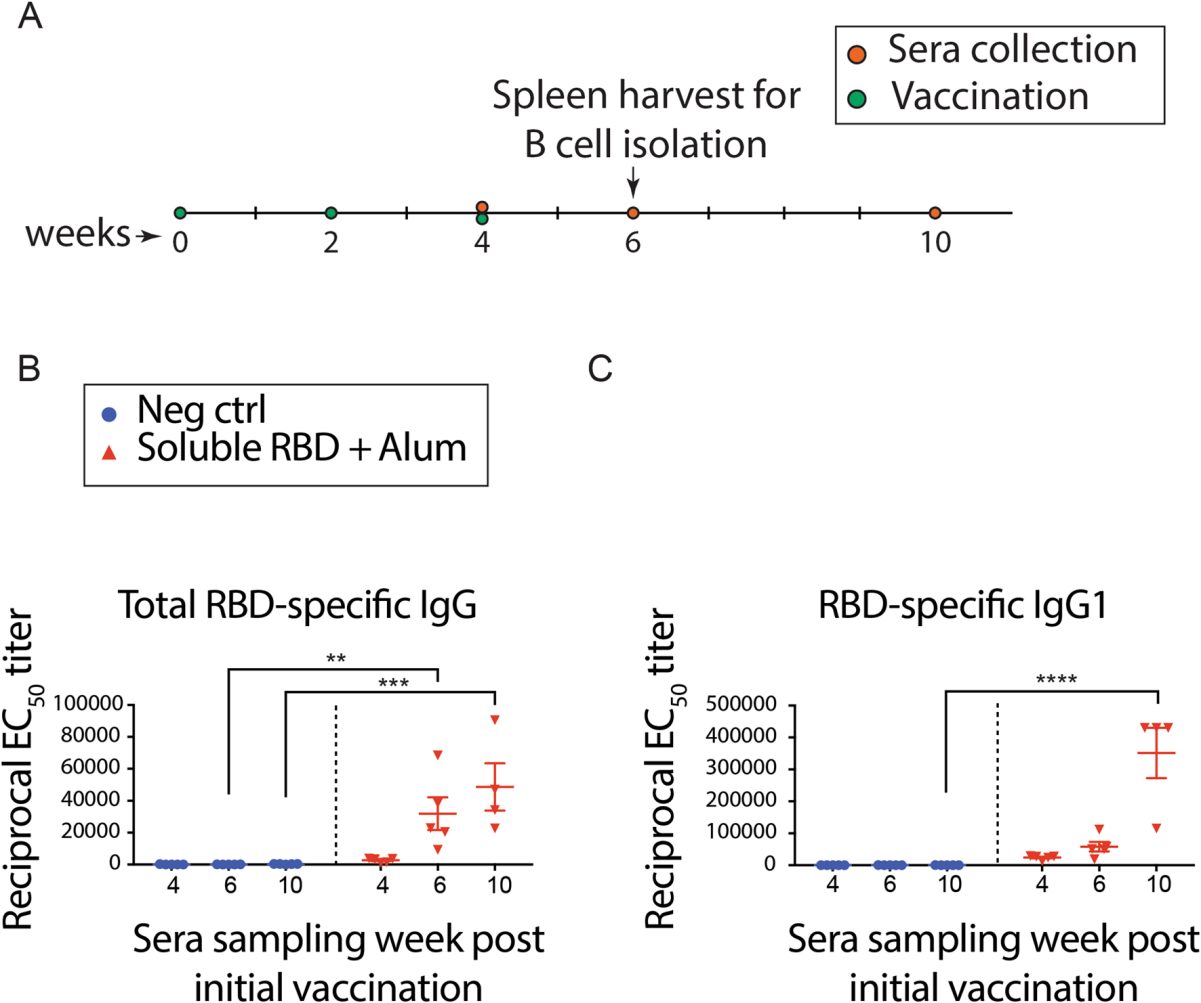
RBD-immunized mice demonstrate significant total RBD-specific IgG and IgG1 responses. (**A**) BALB/c mice were vaccinated three times at 2-week intervals. On weeks 4, 6, and 10, serum samples were collected for total IgG (**B**) and IgG1 (**C**) titer measurements using a RBD-specific ELISA. A subset of mice was used to harvest spleens for B cell isolation on week 6. Data are presented as mean of reciprocal EC_50_ ± SEM. **p < 0.01, ***p < 0.001, and ****p < 0.0001 by two-way ANOVA followed by row comparisons.

To elicit an IgG1^+^ memory B cell response against the RBD, BALB/c mice were vaccinated three times with a two-week interval between doses via subcutaneous injection at the tail base (Fig. 1). Each dose contained 0.5 µg of SARS-CoV-2 RBD and 500 µg of alum. Sera samples were collected from live mice at weeks 4, 6, and 10. To characterize the RBD-specific immune response, sera samples were evaluated for RDB-specific total IgG and IgG1 antibody responses using an RBD-specific ELISA (Fig. 2A,B). Relative to negative control (unvaccinated) mice, RBD-immunized mouse sera showed significant total RBD-specific IgG antibody titer at 6 weeks (p < 0.01) and also at 10 weeks (p < 0.001, by two-way ANOVA followed by row comparisons), as shown in Fig. 2A. Further, RBD-immunized mouse sera exhibited a significant RBD-specific IgG1 antibody response at 10 weeks (p < 0.0001), as shown in Fig. 2B. Together, these data indicate that this vaccination strategy results in significant anti-RBD antibody response, which is first apparent at 6 weeks post-vaccination (RBD-IgG titer), and more significantly corroborated at week 10 (RBD-IgG and IgG1 titer). Moreover, these data support the feasibility of screening for single anti-RBD IgG1^+^ memory B cells at the 6- and 10-week time points.

### Single-cell sorting enables efficient isolation of anti-RBD IgG1^+^ memory B cells

**Figure 3:**
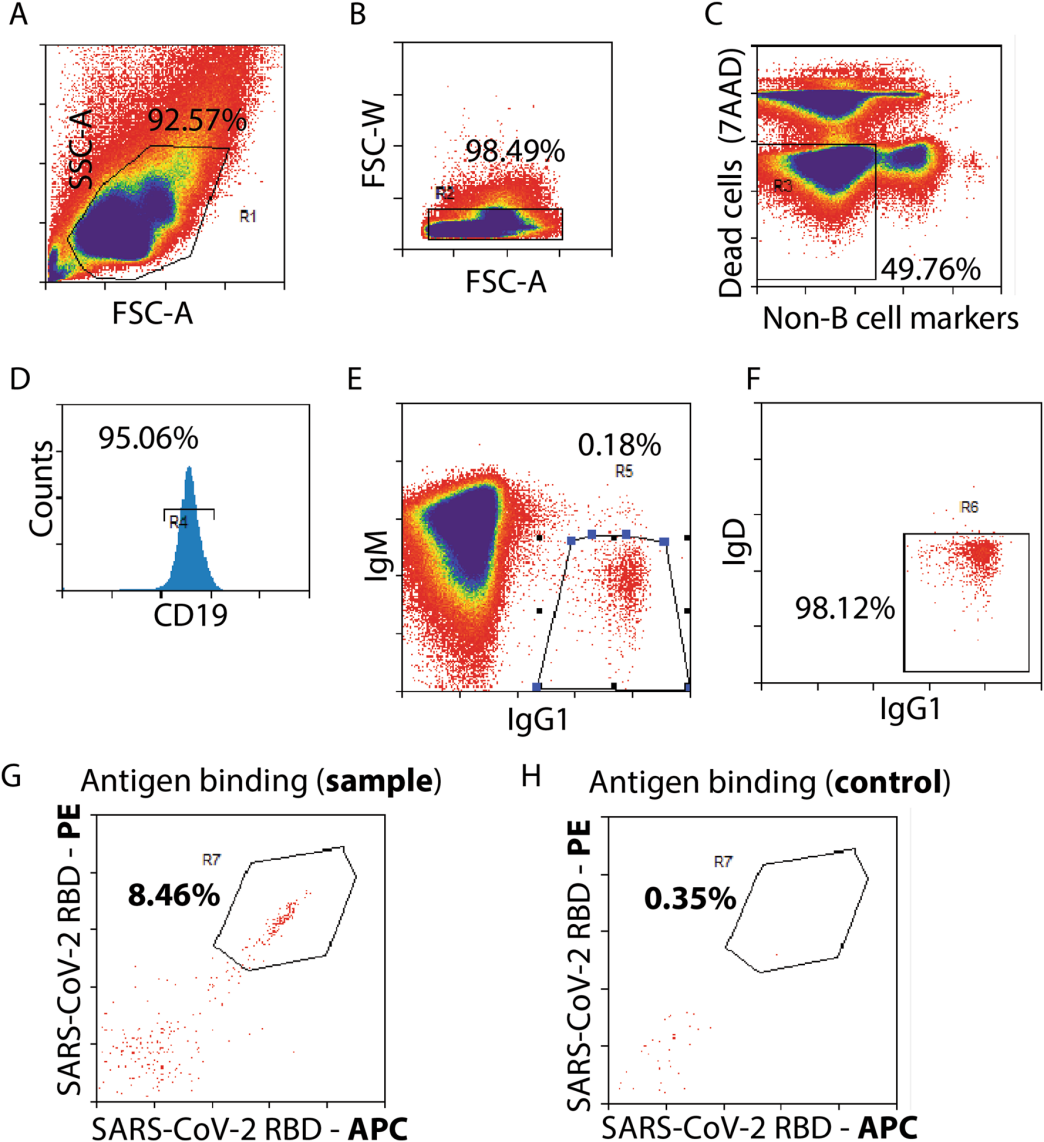
Gating strategy for the isolation of RBD-specific memory B cells via single cell sorting. Flow cytograms of cell samples from (**A**–**G**) immunized BALB/c mice and (**H**) control naïve mice are shown. The following (**A**–**G**) gating strategy was applied in series and gate R7 was sorted as single cells into 96-well plates: (**A**) selection of lymphocytes based on size (forward scatter area) and granularity (side scatter area), (**B**) doublet discrimination based on forward scatter width and forward scatter area, (**C**) viability and lack of non-B cell markers (T cell marker CD4, T cell marker CD8, neutrophil marker GR-1, macrophage marker F480), (**D**) B cell marker CD19, (**E**) negative for IgM (naïve B cell marker) and positive for IgG1, (**F**) negative for IgD (naïve B cell marker), and (**G**) binding to SARS-CoV-2 RBD labeled with PE and APC fluorescent proteins. Flow cytograms for control naïve mice are presented in Supplementary Fig. 1. The SARS-CoV-2 RBD antigen binding cytogram for (**H**) naïve mice is presented for comparison.

Given our focus on the discovery of SARS-CoV-2 neutralizing antibodies, a subset of immunized mice, one vaccinated and one control, were sacrificed at week 6 for single B cell sorting (Fig. 2A). Spleens were excised from immunized and control mice, immediately processed to single cell suspensions of lymphocytes, and further processed to enrich for B cells via magnetic-activated cell sorting (MACS) for CD19^+^ cells. Cell suspensions were subsequently labeled with a panel of detection antibodies (Table S1), as well as two RBD-fluorophore conjugates, namely allophycocyanin (APC) and R-phycoerythrin (PE). A subset of cells were reserved for single color compensation labeling. For single cell sorting, a series of gates were applied to isolate anti-RBD IgG1^+^ memory B cells (Fig. 3A–G). Analysis of the labeled cell suspension showed an abundance of CD19^+^ B cells (Fig. 3D, gate R4 > 95%), suggesting the effectiveness of the MACS-based B cell pre-enrichment strategy. Cells possessing a phenotype of IgG1^+^ and IgM^-^ represented a rare subset of cells (Fig. 3E, gate R5 < 0.5%) with most cellular events demonstrating an IgM^+^ phenotype characteristic of naïve B cells (Fig. 3E). Encouragingly, double positive (RBD-APC^+^ and RBD-PE^+^) IgG1^+^ memory B cells represented 8.46% of events per cytogram in the RBD-immunized sample (Fig. 3G), whereas the equivalent population in the unvaccinated control sample represented only 0.35% of events per cytogram (Fig. 3H). This corresponds to a signal-to-background ratio > 24, suggesting low risk of identifying false-positive cells, namely those with anti-RBD IgG1^+^ memory B cell phenotype (as assessed by our gating strategy), but otherwise lacking anti-RBD antibody expression. The complete set of flow cytograms from control mouse samples are detailed in Supplementary Fig. 1. Based on these results, single cells were sorted into individual wells in 96-well plates, and processed for recovery of V_H_/V_L_ genes.

### Single-cell analysis affords efficient recovery of paired V_H_/V_L_ genes

To evaluate the efficiency of paired V_H_/V_L_ gene recovery, a single 96-well plate containing anti-RBD IgG1^+^ memory B cells (one per well) was probed for V_H_ and V_L_ genes using a series of PCR steps reported previously^20^. The process involved one round of RT-PCR to convert cellular RNA to cDNA, an initial round of PCR to amplify V_H_ and V_L_ (kappa) genes using a primer set specific for common IgG1 murine antibody framework regions, and a second round of PCR to amplify full length V_H_ and V_L_ genes. PCR products were subsequently sequenced via Sanger sequencing. Of the 96 wells evaluated, 18 resulted in recovery of paired V_H_/V_L_ genes, 48 resulted in recovery of V_H_ genes only, and 3 resulted in recovery of V_L_ genes only. In examining resulting paired V_H_/V_L_ genes for replicate sequences, a total of 14 unique antibody pairs were identified. The resulting 14 pairs were cloned into heavy and light chain mammalian expression plasmids containing human IgG1 constant regions and kappa light chain regions for chimeric antibody expression. Antibodies were expressed in HEK 293-6E cells via transient transfection and subsequently purified using Protein A beads, which resulted in the isolation of five lead antibodies (12H2, 13I1, 1A1, 4A7, 6C5) with purification yields of ~ 10–40 mg/L. Lead antibody sequences and phylogenetic analysis are respectively presented in Supplementary Figs. 2 and 3. SDS-PAGE analysis of the lead clones indicated molecular weights consistent with theoretical values (Supplementary Fig. 4). Encouragingly, analytical size-exclusion chromatography (SEC) further validated the predicted antibody sizes and high purities for non-denaturing conditions. Greater than 95% monomer content was observed for all antibodies tested (Supplementary Fig. 5).

### Isolated antibodies demonstrate high affinity for SARS-CoV-2 RBD

**Figure 4:**
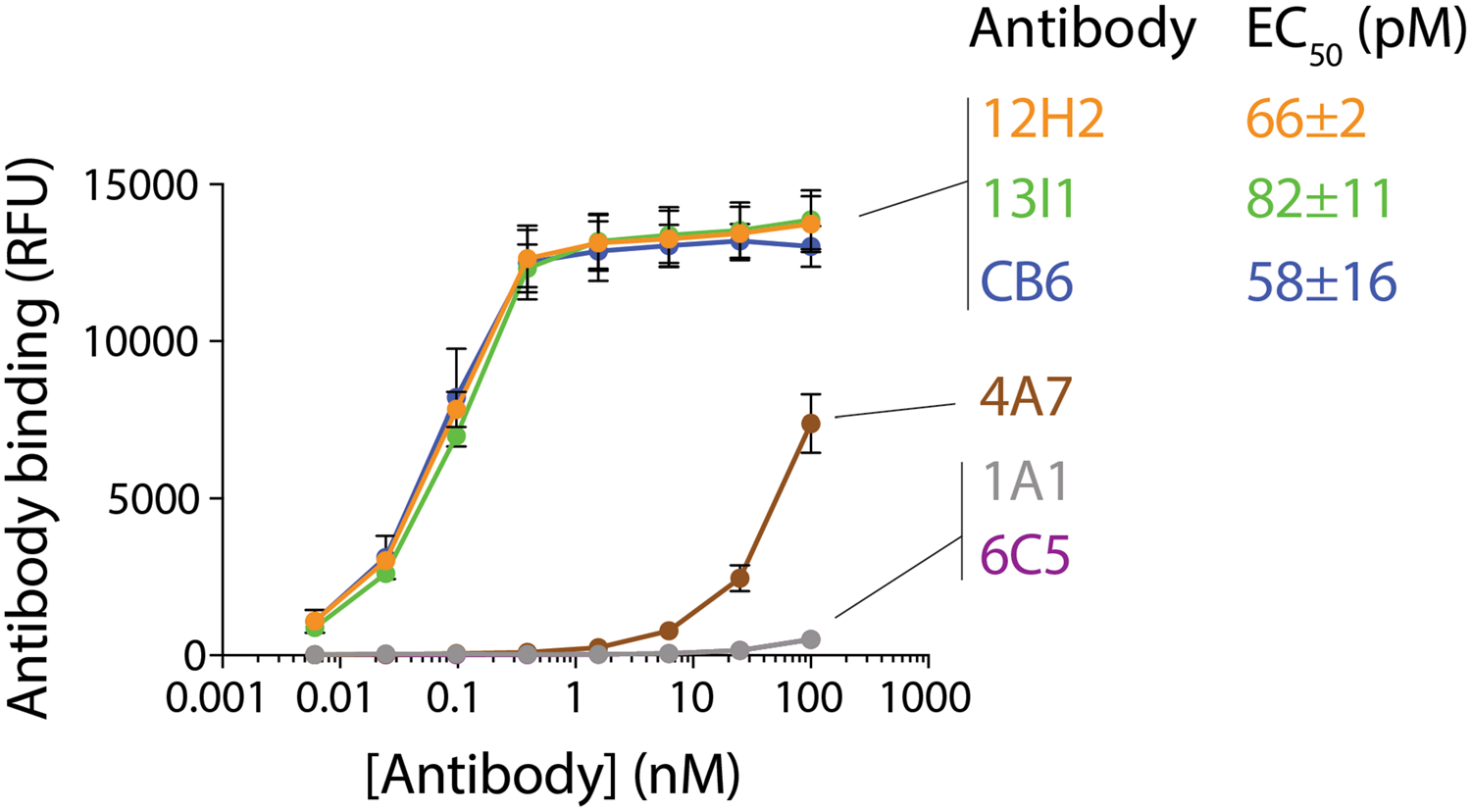
Isolated antibodies possess high affinity for the SARS-CoV-2 receptor-binding domain. Flow cytometry analysis of binding of antibodies to biotinylated SARS-CoV-2 RBD immobilized on magnetic beads was assessed via flow cytometry. Half-maximal effective concentrations (EC_50_) are presented for the high affinity antibodies (12H2, 13I1) and a positive control antibody (CB6). The results are averages of three independent experiments and the error bars are standard deviations.

To evaluate the affinity of lead antibodies to SARS-CoV-2 RBD, bivalent antibodies were co-incubated with biotinylated SARS-CoV-2 RBD immobilized on magnetic beads and binding was assessed via flow cytometry. This affinity analysis revealed that two lead clones (12H2 and 13I1) possess low pM EC_50_ values against RBD (Fig. 4). Fitting of dose-dependent binding response curves indicated that lead clones 12H2 and 13I1 possess EC_50_ values of 66 ± 2 and 82 ± 11 pM, respectively (Fig. 4). For comparison, the previously reported human SARS-CoV-2 antibody CB6, reported to have both high affinity for the RBD and potent neutralizing activity^21^, was expressed and purified in-house and included as a positive control. Encouragingly, our analysis revealed that our potent lead antibodies possess affinity approaching that of CB6 positive control (EC_50_ of 58 ± 16 pM). The other three lead clones (4A7, 1A1, and 6C5) displayed much lower affinities. Overall, these data demonstrate that a subset of the identified antibodies bind SARS-CoV-2 RBD with high affinities.

### Antibodies demonstrate potent neutralizing activity in a SARS-CoV-2 pseudovirus assay

**Figure 5:**
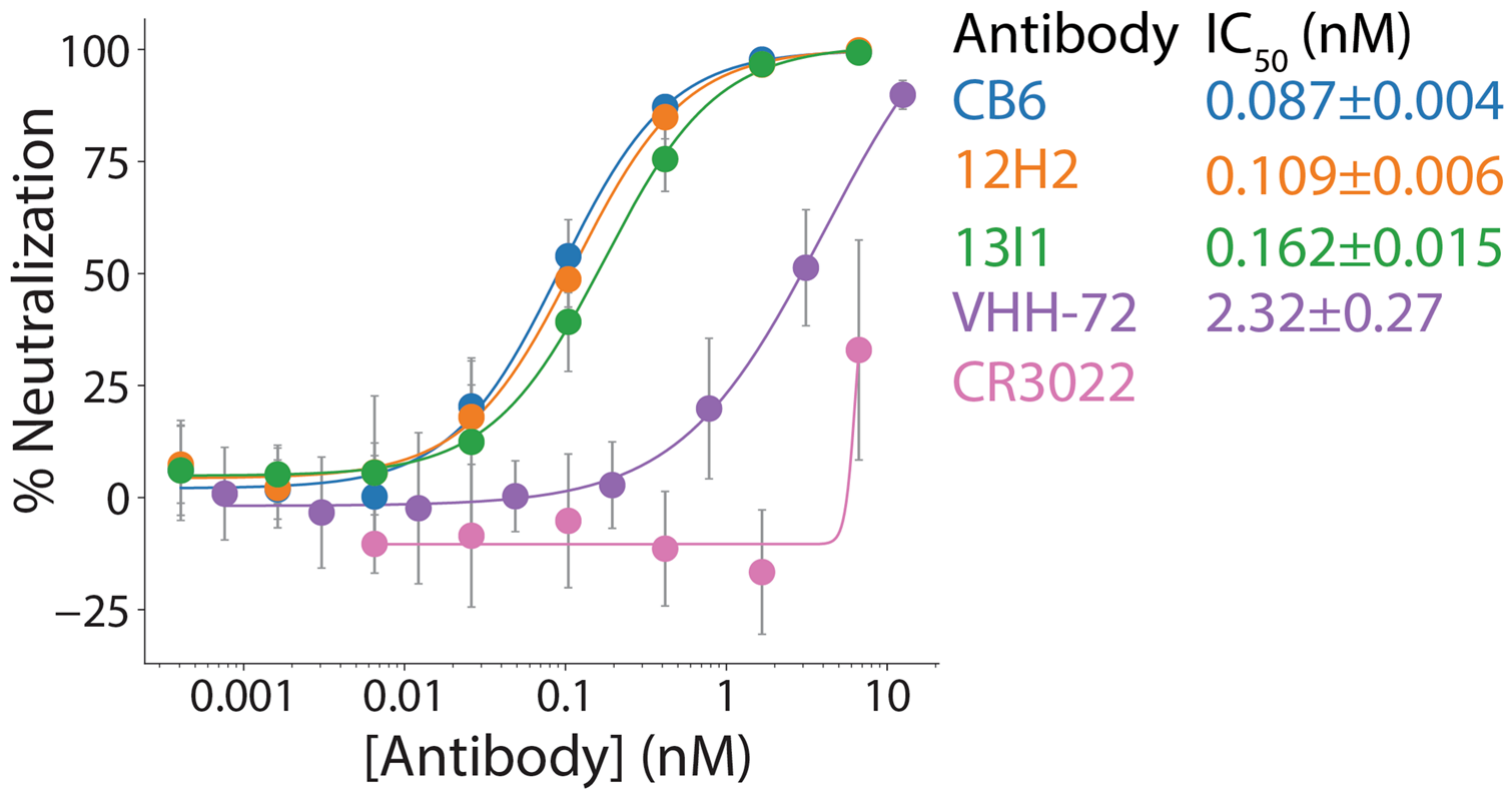
Isolated antibodies demonstrate potent neutralizing activity in a SARS-CoV-2 pseudovirus assay. Neutralizing activities of the high affinity antibodies (12H2 and 13I1) are shown relative to a potent neutralizing control antibody (CB6) and two control antibodies with moderate (VHH-72) and weak (CR3022) neutralizing activity. A lentivirus-based SARS-CoV-2 pseudovirus assay was employed, and the relative luciferase signal was measured 48 h post infection. Half-maximal inhibitory concentrations (IC_50_) values are reported. These data are averages of three repeats, and the error bars are standard deviations.

To evaluate neutralizing activity of the lead antibodies, a lentivirus-based pseudovirus assay was employed that is based on previous work^22^. In this assay, pseudovirus particles, displaying SARS-CoV-2 spike surface protein and containing RNA encoding the expression of luciferase, were co-incubated with antibodies or controls, and then serial dilutions of these mixtures were applied to engineered HEK293T cells stably expressing human ACE2 on the cell surface. Pseudovirus infectivity was quantified in terms of the relative luciferase expression 48 h post-infection. Antibodies 12H2 and 13I1 were evaluated relative to the control neutralizing antibody CB6, as well as two additional controls that were discovered against SARS-CoV with cross-reactivity against SARS-CoV-2. These include VHH-72, a SARS-CoV-2 neutralizing nanobody^23^, and CR3022, a high affinity anti-SARS-CoV-2 monoclonal antibody with limited neutralizing activity^24,25^. As shown in Fig. 5, high-affinity antibodies 12H2 and 13I1 possess potent neutralizing activity. The IC_50_ values were 0.109 ± 0.006 and 0.162 ± 0.015 nM for 12H2 and 13I1, respectively. Similarly, the positive control neutralizing antibody CB6 had an IC_50_ of 0.087 ± 0.004 nM. 12H2 and 13I1 demonstrated significantly improved IC_50_ values relative to VHH-72 (IC_50_ of 2.32 ± 0.27 nM) and CR3022 (IC_50_ > 6.7 nM). Overall, these data demonstrate that the novel RBD-specific antibodies possess potent sub-nM neutralizing activity. Given high sequence similarity of the top performing antibodies 12H2 and 13I1 (V_H_ = 99.2% similarity, V_L_ = 99.1% similarity, Supplementary Fig. 2), subsequent investigation was conducted only with 13I1.

### Potent neutralizing antibody competes with ACE2 for SARS-CoV-2 RBD binding

**Figure 6:**
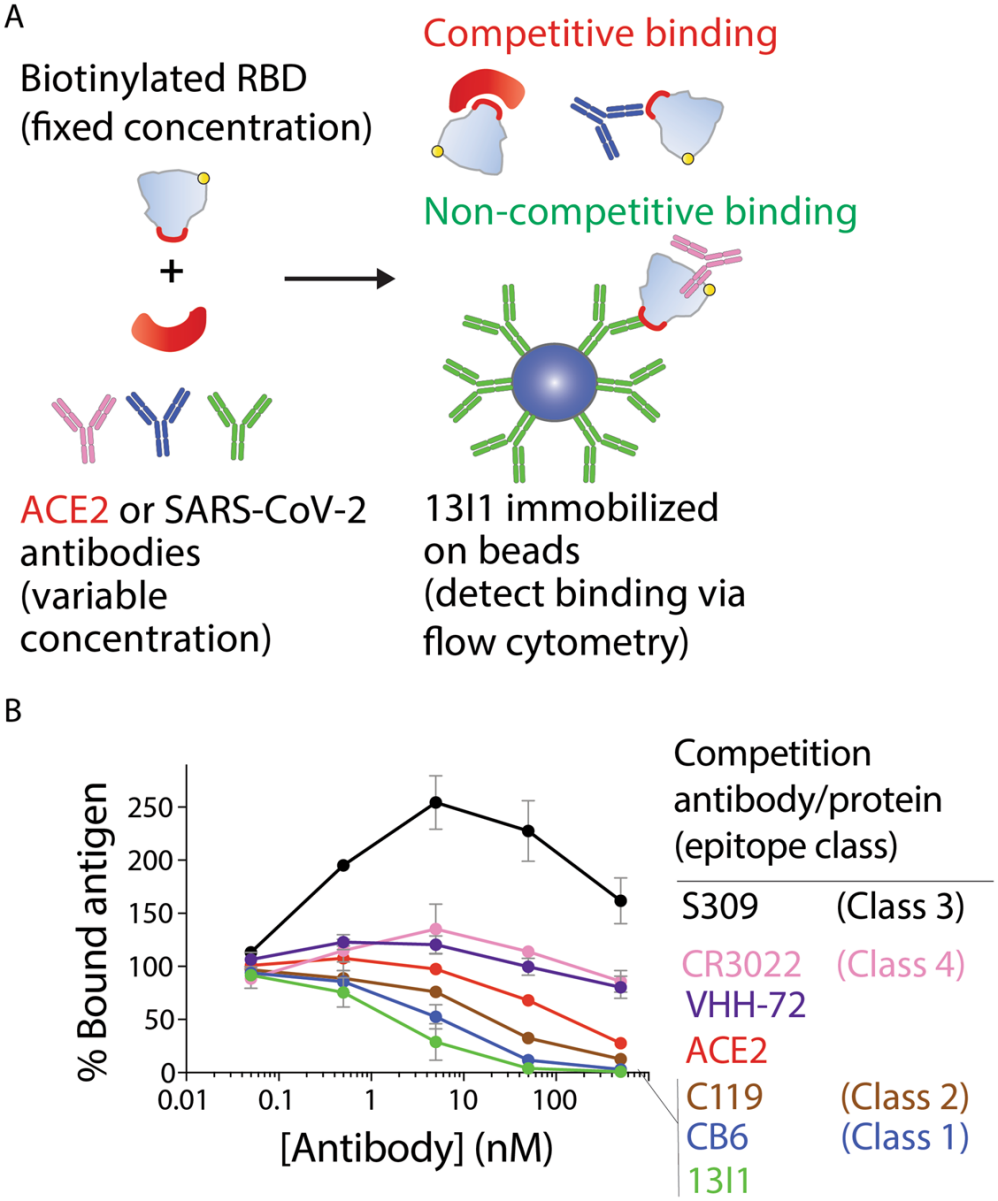
Neutralizing antibody 13I1 competes with ACE2 for binding to the SARS-CoV-2 receptor-binding domain. (**A**) Schematic of competition analysis between 13I1, ACE2 and other antibodies (CR3022, CB6, VHH-72, C119, S309). Competitive binding analysis was employed in which 13I1, reference antibodies or ACE2 was pre-incubated with biotinylated RBD (5 nM) over a range of concentrations of antibodies or ACE2, and then co-incubated with immobilized 13I1 on Protein A magnetic beads. (**B**) The percentage of SARS-CoV-2 RBD bound is reported relative to the amount bound in the absence of pre-blocking with antibodies or ACE2. The results are averages from two independent experiments and the error bars are standard deviations.

To evaluate the antibody binding epitope and mechanism of neutralization, competitive binding analysis was employed in which antibodies (13I1, CB6, CR3022, VHH-72, C119, S309) or human ACE2 were pre-incubated with biotinylated SARS-CoV-2 RBD (5 nM) over a range of antibody or ACE2 concentrations, and then co-incubated with 13I1 immobilized on Protein A magnetic beads (Fig. 6). The percentage of RBD bound to 13I1-coated beads, as evaluated by flow cytometry, is reported relative to the amount of RBD bound in the absence of competitor. As expected, incubation of the RDB with 13I1 resulted in near complete inhibition of RBD binding to 13I1-coated beads. Notably, RBD pre-incubation with ACE2 resulted in partial inhibition of 13I1 engagement with RBD. Mechanistically, this suggests that the neutralizing activity of 13I1 is at least in part attributed to inhibition of RBD engagement with human ACE2.

Importantly, competition analysis was conducted with previously reported SARS-CoV-2 antibodies^26^ that engage unique epitopes on the SARS-CoV-2 spike protein: CB6 (class 1), C119 (class 2), S309 (class 3), CR3022 (class 4). Interestingly, CB6 antibody, which showed similar high affinity and neutralizing activity relative to 12H2 and 13I1, was observed to be strongly competitive with 13I1 for RBD binding. Similarly, C119 antibody was also observed to compete strongly with 13I1 for RBD binding, suggesting that 13I1 recognizes a partially overlapping or proximal epitope on the RBD relative to that for both CB6 (class 1) and C119 (class 2). Conversely, S309 did not compete with 13I1 for RBD binding. Interestingly, 13I1 binding to RBD was markedly enhanced in the presence of S309, suggesting that engagement of the RBD at an epitope characteristic of class 3 antibodies may stabilize 13I1 binding to a non-competitive RBD epitope. Further, the CR3022 antibody and VHH-72 nanobody were also not competitive with 13I1 for RBD binding, suggesting that 13I1 recognizes a unique epitope relative to these controls.

### Increased valency enhances 13I1 neutralizing activity

**Figure 7:**
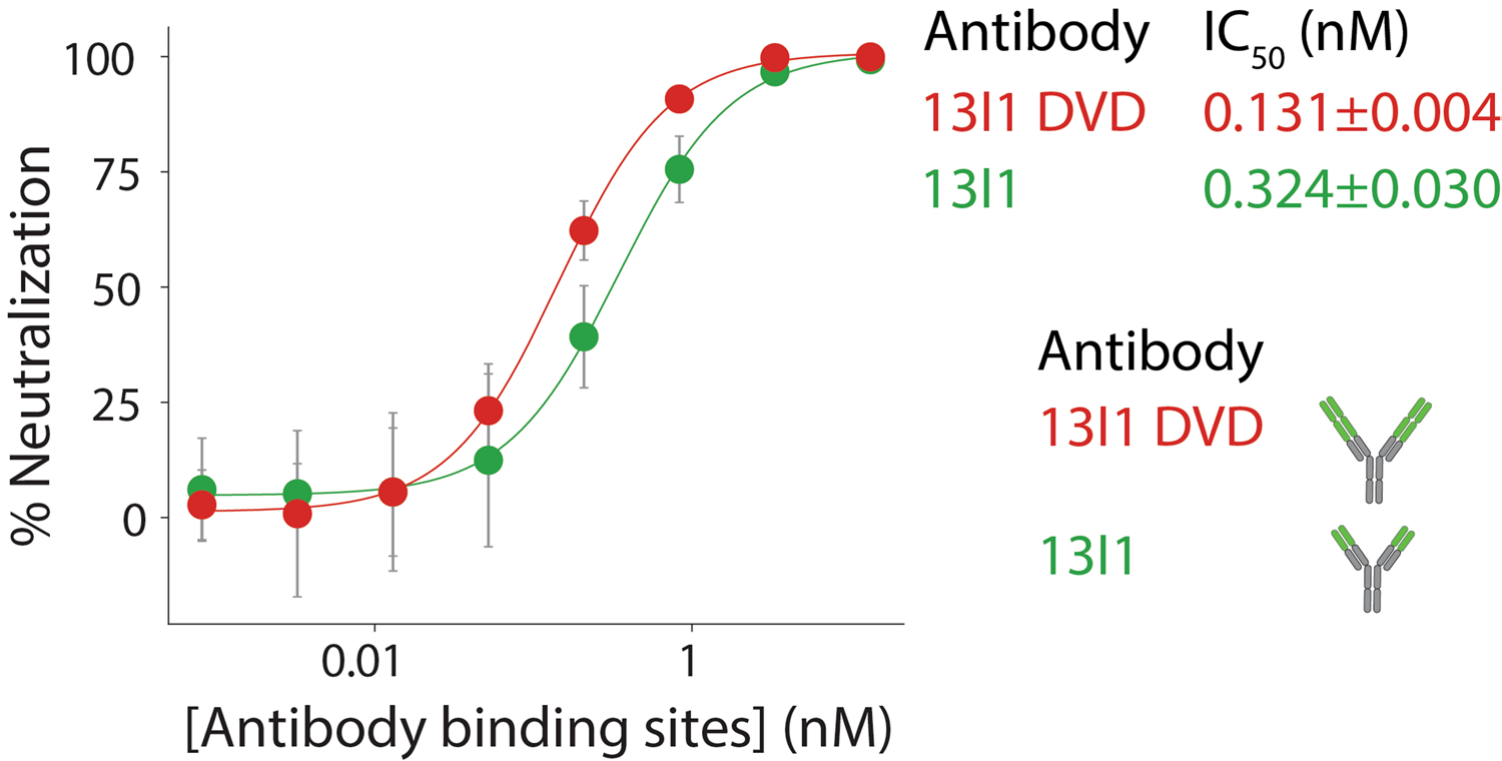
Increased valency enhances 13I1 neutralizing activity. Neutralizing activity of tetravalent 13I1 DVD-formatted antibody is shown relative to 13I1 bivalent antibody. A lentivirus-based SARS-CoV-2 pseudovirus assay was employed, and the relative luciferase signal was measured 48 h post infection. Calculated half-maximal inhibitory doses (IC_50_) are presented. 13I1 DVD neutralizing activity was evaluated in parallel with 13I1 data collected in Fig. 5 and is shown here for clarity. These data are averages of three independent repeats, and the error bars are standard deviations.

Next, we sought to investigate the impact of valency on antibody neutralizing activity. Toward this goal, we engineered a tetravalent dual-variable domain (DVD) version of the 13I1 antibody and directly evaluated the neutralizing activity relative to the bivalent 13I1 IgG via the SARS-CoV-2 pseudovirus assay (Fig. 7). Antibodies were compared on the basis of binding site concentration to account for differences in valency. Encouragingly, 13I1 DVD showed enhanced neutralizing activity (IC_50_ of 0.131 ± 0.004 nM) relative to bivalent 13I1 mAb (IC_50_ of 0.324 ± 0.030 nM) even after accounting for valency differences. These data demonstrate that increasing valency synergistically enhances the neutralizing activity of the 13I1 antibody.

### Neutralizing antibodies possess high specificity and stability

**Figure 8:**
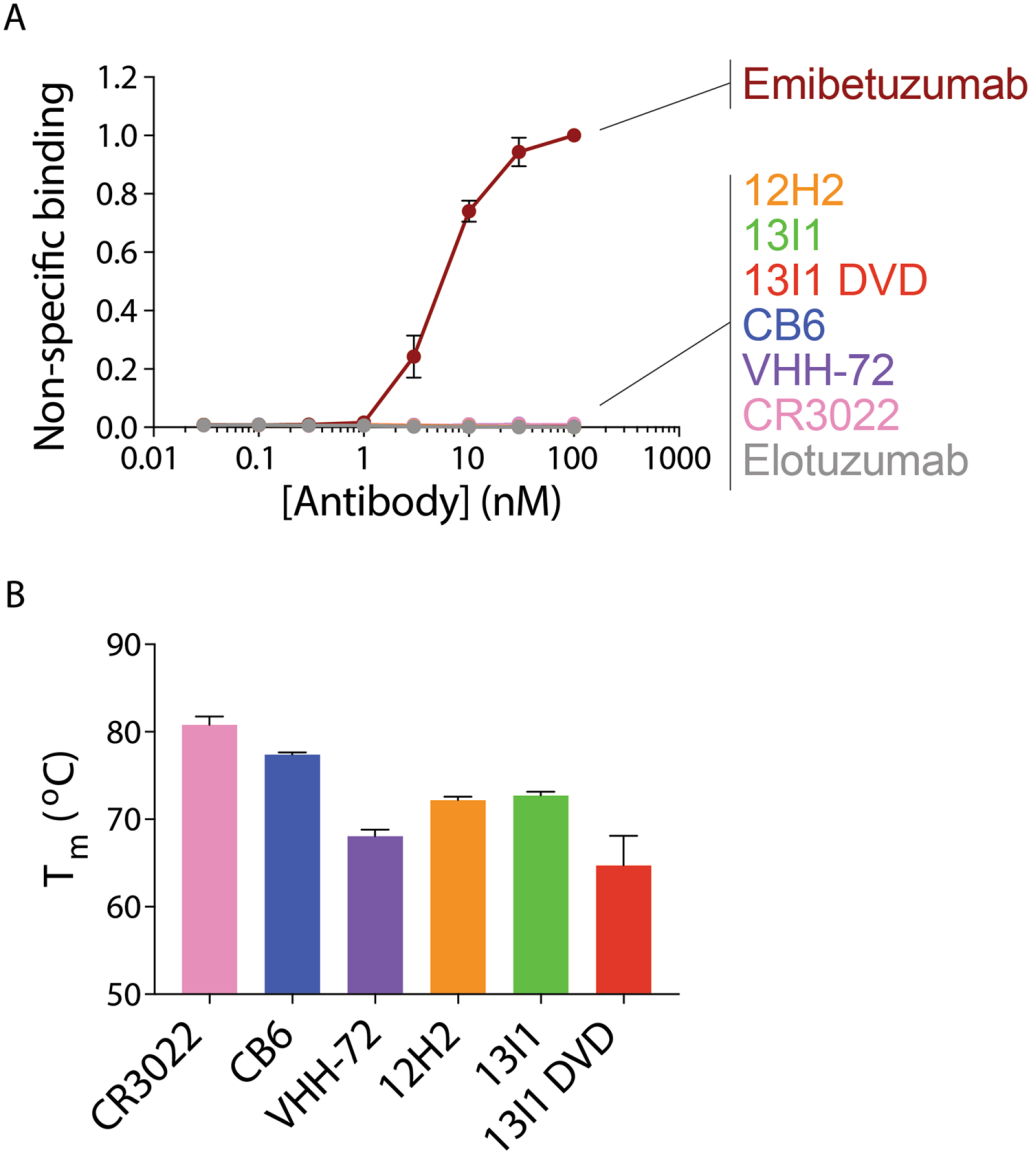
Neutralizing antibodies possess high specificity and stability. (**A**) Non-specific binding of antibodies (immobilized on Protein A magnetic beads) was evaluated via incubation with biotinylated soluble membrane proteins from CHO cells and detection via flow cytometry. Control antibodies with high (emibetuzumab) and low (elotuzumab) non-specific binding were also evaluated for comparison. The two control antibodies are not identical to the actual drugs, as they have the variable regions of the actual drugs and a common IgG1 framework. (**B**) Melting temperatures of antibodies were evaluated via differential scanning fluorimetry. Results are averages from two independent experiments and the error bars are standard deviations.

We next sought to characterize the biophysical properties of the most neutralizing antibodies identified in this work (Fig. 8). Toward this goal, we conducted non-specific binding analysis via incubation of antibodies with a polyspecificity reagent that consists of biotinylated soluble membrane proteins derived from CHO cells^27,28^. The relative binding of the biotinylated soluble membrane proteins to each antibody immobilized on Protein A magnetic beads was evaluated using flow cytometry (Fig. 8A). Control clinical-stage antibodies with high (emibetuzumab) and low (elotuzumab) levels of non-specific binding were also evaluated for comparison. Encouragingly, the antibodies generated in this work (12H2, 13I1, 13I1 DVD) showed extremely low levels of non-specific binding, comparable to that of elotuzumab. We further evaluated antibody stability in terms of the melting temperature (*T*_*m*_) via differential scanning fluorimetry. The control antibodies displayed *T*_*m*_ values of 80.8 ± 0.8 °C (CR3022), 77.4 ± 0.2 °C (CB6), and 68.1 ± 0.6 °C (VHH-72; Fig. 8B). The IgGs discovered in this work were observed to have *T*_*m*_ values of 72.2 ± 0.3 °C (12H2), 72.7 ± 0.4 °C (13I1) and 64.7 ± 3.0 °C (13I1 DVD). The 13I1 DVD antibody exhibited an unfolding profile containing two local maxima, indicating multiple unfolding transitions, whereas the other antibodies exhibited a single unfolding transition. Overall, these data highlight the favorable specificities and stabilities of the antibodies generated in this work.

## Discussion

Previous reports indicate single B cell screening can be employed to isolate high-affinity antibodies as quickly as within one week (from single B cell isolation to lead molecule evaluation), which is highly attractive for applications against infectious diseases^8–13^. The rate-limiting step in the overall process is the time required to elicit an antibody response. Here, we show that potent neutralizing antibodies can be isolated from BALB/c mice six weeks post vaccination. Our RBD-specific ELISA results (Fig. 2) suggest significant opportunity for single B cell screening at ten weeks post vaccination as well, although this was not evaluated here. We further demonstrate that mouse antibody genes can be directly cloned into mammalian expression plasmids for expression and purification of chimeric antibodies (mouse variable and human IgG1 constant regions), which possess potent neutralizing activity. Previous work illustrates an alternative approach, referred to as transcriptionally-active PCR (TAP), which can also be employed for improved throughput and accelerated timelines^8,9^. This TAP method allows for expression of lead antibodies without the need for subcloning into expression cassettes, and may be attractive for large scale, single B cell discovery campaigns.

Notably, we anticipate that the antibodies described herein may hold value for immediate practical use, including diagnostic applications and tools for study in transgenic murine models (expressing human ACE2) of SARS-CoV-2^29–35^. While the use of BALB/c mice as a host species carries inherent limitations for the development of therapeutically-relevant antibodies without humanization, the methods described herein are easily amenable to the screening of human-derived antibodies using samples obtained from humans or humanized mice. A modified protocol is required that includes alternative detection antibodies and PCR primer sets, and is detailed elsewhere^10,12^.

Our work along with other previous studies^14,36–38^ motivate the continued exploration of isolating SARS-CoV-2 antibodies after vaccination, especially from human samples for therapeutic applications. Antibodies generated in this work possessed high affinity (low pM EC_50_ values in the bivalent format), which suggests in vivo affinity maturation following six weeks from the initial round of vaccination. Relative to sourcing antibodies from SARS-CoV-2 infected subjects, evidence suggests that vaccination may result in superior immune responses with regard to antibody affinity maturation via somatic hypermutation and long-lived memory B cell responses^36,39–41^. For example, spleen and lymph node tissues isolated *post mortem* from infected humans lack germinal centers, indicating incomplete affinity maturation and insufficient memory response^36^. This and other studies^36–41^ suggest that vaccinated subjects may be a rich source for the isolation of antibodies with high affinity and potent neutralizing activity, and the time of B cell sampling after vaccination should be carefully considered to optimize the antibody isolation process.

Our investigation could be logically extended for robust response to COVID-19 and other emergent infectious diseases. The single B cell sorting strategy employed here was specifically designed to mitigate risk of false-positive lead identification, and thereby focus investigation on cells most likely to possess high-affinity antibodies. This was accomplished by employing a double-positive selection strategy, using antigen separately labeled with two unique fluorophores (Fig. 3G). This strategy was informed by a previous report in which antibodies against tumor necrosis factor receptor 2 were identified with low pM affinities^9^. In this previous work, flow cytograms suggest some degree of non-specific binding, illustrating clear rationale for this refined antigen selection strategy^9^. Interestingly, in our work, non-specific antigen binding was low, which is illustrated by flow cytograms from both immunized and control mouse spleen samples (Fig. 3G,H) and the rarity of single-positive cells (either RBD-PE^+^ or RBD-APC^+^ cells). Overall, this finding indicates that the degree of non-specific antigen binding may vary depending on the specific antigen that is employed.

Provided that non-specific antigen binding is of minimal concern (at least in this study), single B cell screening may enable efficient selection of cells that simultaneously engage multiple different antigens. For example, an extension of our general approach may enable the rapid generation of potent neutralizing antibodies that possess broad neutralizing activity. Specifically, the gating strategy could be modified to screen for double-positive cells that bind RBDs of both SARS-CoV-2 and SARS-CoV. The antibodies described in this work show high affinity for SARS-CoV-2 but lack affinity for SARS-CoV (Supplementary Fig. 6), indicating that 12H2 and 13I1 engage an epitope on SARS-CoV-2 that is not conserved in SARS-CoV. While tradeoffs between affinity and neutralizing activity may complicate isolation of broadly neutralizing antibodies, single B cell sorting may afford a facile approach toward this goal. In another similar proposed extension of this work, single B cell cloning may also enable screening for B cells possessing a double-positive binding phenotype against wild-type and highly infectious SARS-CoV-2 variants. This proposal is motivated by multiple reports suggesting the emergence of SARS-CoV-2 mutant variants that may escape engagement by SARS-CoV-2 antibodies^42–44^. Encouragingly, 13I1 was observed to retain binding to the SARS-CoV-2 S1 protein of the United Kingdom variant (B.1.1.7). However, binding activity was largely abrogated against the S1 protein of the South African variant (B.1.351), as shown in Supplementary Fig. 7. This finding is consistent with other reported antibodies and nanobodies^45,46^ that compete with class 1 and class 2 antibodies for RBD binding, and may provide valuable insight toward the informed future generation of broadly neutralizing antibodies.

Beyond exquisite control of the antigen presentation and concentration during FACS-based cell screening, single B cell cloning is also attractive given the control that is afforded at the stage of vaccination. Future investigation may be warranted for single B cell screening from mice vaccinated with optimized antigen concentrations and alternative and optimized vaccine formulations. For example, the relatively low amount of RBD per injection (0.5 µg/dose) may have contributed to low titers on week 4. However, we observed robust sero-conversion by week 6. For comparison, other SARS-CoV-2 vaccine studies with RBD have used significantly higher amounts of protein^47–49^. To further inform vaccine design, a logical extension of this work may be to evaluate the impact of SARS-CoV-2 antigen (*e.g.,* RBD, S1, S1 trimer) as well as adjuvant formulation on the resulting antibody responses. To identify the most promising candidates for single B cell sorting, serum samples could be analyzed for both antibody response via an antigen-specific ELISA and neutralizing activity via the SARS-CoV-2 pseudovirus assay. Furthermore, antibody isotype and subclass are also important factors for consideration in future work. The antibodies isolated in this work are IgG1s given that anti-mouse IgG1 detection antibodies were employed for positive selection in our gating strategy. The majority of the selection strategies in previous reports also focus on IgG1 antibodies^9–11,20^. Nevertheless, further investigation may be warranted for single B cell screening in a manner that does not bias selection toward IgG1 antibodies, especially in settings in which other subclasses are upregulated or predominant.

Future studies should consider and address several potential pitfalls related to our studies. First, the use of inbred mice (as done in this study) may be suboptimal for B cell maturation relative to outbred mice given that inbred mice are associated with reduced MHC class II diversity compared to outbred mice^50,51^. Nevertheless, many vaccine studies, including SARS-CoV-2 studies^52–55^, have used inbred mice (e.g., BALB/c mice) for proof-of-concept data prior to clinical translation. Second, a final intravenous antigen injection 3–4 d prior to splenectomy (not employed in this study) should likewise be considered. When the goal is to generate hybridomas, it is common to use a booster injection after suitable titers are reached^56,57^, which is implicated in the migration of circulating B cells to the spleen. Nevertheless, other single B cell antibody discovery campaigns report a similar dosing regimen as we used^9^. Future investigation is warranted to further characterize the affinities of the antibodies discovered herein (e.g., by SPR) and evaluate the neutralizing activity of these antibodies against live SARS-CoV-2 virus and pseudovirus for CDC variants of concern. Future efforts in optimizing the immunization and B cell screening process are expected to continue to advance the generation of antibodies with high affinity and specificity, which is especially important for addressing current and future pandemics.

## Conclusions

We have demonstrated the use of single B cell screening for the discovery of high-affinity, potent neutralizing antibodies against SARS-CoV-2. This work highlights a facile approach for the rapid discovery of antibodies to address infectious diseases such as COVID-19. We expect that the methodologies applied here can be readily extended to other highly infectious SARS-CoV-2 variants and emergent infectious diseases.

## Materials and methods

### Production of recombinant RBD

SARS-CoV-2 RBD was produced in HEK293T cells from a clone kindly provided by Prof. Florian Kramer (Icahn School of Medicine at Mt. Sinai) and purified as reported^58^ with a slight modification to purify the monomeric form of RBD used in this study. The protein was further purified on a Superdex 75 column (Cytiva) pre-equilibrated with phosphate buffered saline.

### In vivo vaccination study

Animals were cared for following federal, state, and local guidelines. The University of Michigan, Ann Arbor is an AAALAC international accredited institution, and all works conducted on animals were in accordance with and approved by the Institutional Animal Care and Use Committee (IACUC). All animal experiments were carried out in compliance with the ARRIVE (Animal Research: Reporting of In Vivo Experiments) guidelines. Female BALB/c (n = 5 per group) mice 5–6 weeks of age were purchased from Jackson Laboratory. Mice were used without further randomization. Mice (n = 5 per group) were given a week of acclimation and were vaccinated 3 times with a 2-week interval. Each dose containing 0.5 µg of RBD and 500 µg of alum (Alhydrogel, Invivogen) was subcutaneously injected at the tail base. Immune sera were collected on weeks 4, 6, and 10 and analyzed for RBD-specific IgG and IgG1 antibody titers by ELISA. Briefly, RBD protein was coated on 96-well ELISA plates (0.1 µg/well), and serially diluted sera samples were added. After an hour of incubation and multiple washings, horseradish peroxidase (HRP)-labeled secondary antibodies were added and incubated for 1 h at room temperature. Secondary antibodies used were rabbit anti-mouse IgG H&L-HRP (Abcam) and goat anti-mouse IgG1-HRP (Southern Biotech). TMB substrate solution was added, and the reaction was stopped by the addition of NaF. The absorbance was measured at a 620 nm wavelength using a plate reader (Synergy Neo, BioTek). To measure antibody titers, titration curves were evaluated based on the absorbance and the dilution factor, from which half maximal effective concentration (EC_50_) values were calculated using software Gen5 (BioTek). A subset of mice was used for spleen harvest on week 6.

### B cell isolation and staining

To isolate B cells, freshly excised mouse spleens were washed with ice-cold FACS buffer (HBSS no calcium or magnesium with 1 mM EDTA, 25 mM HEPES, 1% FBS) and processed through a 70 µm cell strainer. Cells were washed once with ice-cold FACS buffer, and then treated with AKC lysis buffer for 2 min to lyse red blood cells. Cells were then washed again with FACS buffer and passed through a 40 µm cell strainer. Magnetic-activated cell sorting (MACS) was then performed to enrich for B cells based on CD45R expression. Briefly, splenocytes were incubated with mouse FcR blocking reagent (Miltenyi Biotec, 130-092-575) following the manufacturer’s protocol. Splenocytes were then incubated with mouse CD45R (B220) microbeads (Miltenyi Biotec, 130-049-501) and MACS was performed following the manufacturer’s protocol using LS columns (Miltenyi Biotec, 130-042-401) and a Midi MACS separator (Miltenyi Biotec, 130-042-302).

Enriched cells were counted and prepared for fluorescence-activated cell sorting (FACS) by adding a mixture of fluorescently labeled antibodies and antigen. Labeling antibodies were added at 1:1000 dilution in a volume of 1 mL per 10^7^ cells for 30 min at room temperature. The following labeling scheme was employed, listed in the format of ‘molecular target (fluorophore)’: CD19 (AF700), IgG1 (BV421), CD4 (FITC), CD8 (FITC), GR-1 (FITC), F4/80 (FITC), IgM (PE Cy7). SARS-CoV-2 RBD-PE and RBD-APC were each added at 1 µg per 10^8^ cells. Prior to the assay, SARS-CoV-2 RBD (Acro Biosystems, SPD-C52H3) was labeled using Lightning Link PE and APC labeling kits (Novus Biologics, 705–0030 and 703–0030) following the manufacturer’s protocol. Cells (5 × 10^5^) were reserved for each single color compensation control, and labeling antibodies and RBD antigen were likewise applied at 1:1000 dilution for 30 min at room temperature. Following the antibody labeling/antigen incubation step, cells were washed twice with cold FACS buffer. Dead cell marker 7-Aminoactinomycin D (7-AAD) (Invitrogen, number A1310) was added 10 min prior to cell sorting.

The following labeling antibodies were used for FACS preparation: rat anti-mouse IgG brilliant violet 421 (Clone A85-1; BD biosciences, 562580), rat anti-mouse IgM PE-Cy7 (PE/Cy7 anti-mouse IgM Antibody Clone RMM1; Bio legend, 406513), rat anti-mouse IgD APC-Cy7 (APC/Cy7 anti-mouse IgD Antibody, Clone 11-26c; BioLegend, 405715), rat anti-mouse CD19 AF700 (Alexa Fluor® 700 Rat anti-Mouse CD19, Clone 1D3; BD biosciences, 557958), rat anti mouse CD4 FITC (FITC Rat Anti-Mouse CD4, Clone GK1.5; BD Biosciences, 557307), rat anti mouse CD8 FITC (FITC Mouse Anti-Rat CD8a, Clone OX-8; BD Biosciences, 561965), rat anti mouse GR1 FITC (FITC Rat Anti-Mouse Ly-6G and LY-6C, Clone RB6-8C5; BD Biosciences, 553126), rat anti mouse F4/80 FITC (F4/80 Monoclonal Antibody BM8, FITC, eBioscience; Thermo Fisher, 11-4801-82).

The following labeling antibodies were used for single color compensation controls: rat anti mouse CD45—Brilliant violet 421(Clone 30-F11; BD biosciences, 563890), rat anti mouse CD45—PE Cy7 (Clone 30-F11; BD biosciences, 561868), rat anti mouse CD45—APC Cy7 (Clone 30-F11; BD biosciences, 561037), rat anti mouse CD45—AF700 (Clone 30-F11; BD biosciences, 560510), rat anti mouse CD45—FITC (Clone 30-F11; BD biosciences, 553080), rat anti mouse CD45—PE (Clone 30-F11; BD biosciences, 553081), rat anti mouse CD45-APC (Clone 30-F11; BD biosciences, 553092).

### Mouse single B cell sorting

Antigen-specific IgG1^+^ memory B cells were sorted single cell per well into skirted 96-well plates containing 4 µl of cell lysis solution^20^ using a BD Moflo Astrios Cell Sorter. The following gating strategy was applied in series: selection of lymphocytes based on size (forward scatter area) and granularity (side scatter area), doublet discrimination based on forward scatter width and forward scatter area, viability and lack of non-B cell markers (T cell marker CD4, T cell marker CD8, neutrophil marker GR-1, macrophage marker F4/80), B cell marker CD19, negative for IgM (naïve B cell marker) and positive for IgG1, negative for IgD (naïve B cell marker), and binding to SARS-CoV-2 RBD labeled with PE and APC fluorescent proteins.

### cDNA generation and PCRs

After sorting single B cells into a 96-well plate containing 4 μL of lysis buffer in each well, reverse transcription was performed by adding 7 μL of RT mix I (Table S2) to each well and incubating on a preheated thermocycler (65 °C) for 5 min^20^. The plates were then placed on ice for 5 min and 7 μL of RT mix II (Table S2) was added to each well. The plates were incubated on a thermocycler and were run under the following program to generate cDNA (Table S3). To dilute cDNA, 10 μL of nuclease-free water was added to each well. First, PCR amplification was performed for variable heavy and light chains in separate PCR reactions by mixing 38 μL of PCR mix I (Table S4) with 4 μL of cDNA. Amplification was performed on a thermocycler following the first PCR program (Table S4). A second PCR amplification step was performed by mixing 38 μL of seq-PCR mix (Table S5) with 4 μL of PCR product from the first amplification on a thermocycler following the seq-PCR program (Table S5). Seq-PCR product from each well was loaded onto an agarose gel (2% w/v) and run at 120 V for 30 min. The expected bands (450–500 bp) were purified, and PCR products were sent for Sanger sequencing using appropriate reverse primers for heavy and light chains.

### Cloning and recombinant antibody expression

Antibody V_H_ genes were digested with EcoRI-HF (New England Biolabs, R3101L) and NheI-HF (New England Biolabs, R3131L), whereas V_L_ genes were digested with EcoRI-HF and BsiWI-HF (New England Biolabs, R3553L) and purified (Qiagen, 28104). Expression plasmids (V_H_ or V_L_) were digested with appropriate restrictions enzymes, following the manufacturer’s protocol, and subsequently treated with calf intestinal alkaline phosphatase (New England Biolabs, M0525L). The digested vector was analyzed by electrophoresis using a 1% agarose gel. Appropriate sized DNA was excised and purified. Digested inserts and vectors were ligated with T4 ligase (New England Biolabs, M0202L) and transformed via heat shock into DH5α chemically competent cells. Cells were incubated in the presence of LB media (antibiotic free) for 1 h at 37 °C (shaking at 200 rpm), and then plated on LB plates supplemented with ampicillin (100 µg/mL) overnight at 37 °C. Single colonies were picked, grown in LB media with ampicillin overnight, miniprepped (Qiagen, 27106), and sequenced via Sanger sequencing. The plasmids for the dual variable domain tetravalent antibody were similarly cloned, expressed, and purified.

The antibodies used in this study were expressed in HEK293-6E cells from National Research Council of Canada. The antibody heavy and light chain plasmids (7.5 μg each) were mixed with PEI (45 μg) at room temperature with F17 media (without supplements) for 10–15 min and added to cells at a density of 1.5–2 million cells per mL. Cell media was enhanced with 20% w/v yeastolate (BD Sciences, 292804) 24–48 h post transfection. Cells were grown for an additional 5 days at 37 °C in F17 media containing supplements: Glutamine (Invitrogen, 25030081), Kolliphor (Thermo Fisher Scientific, NC0917244) and G418 (Thermo Fisher Scientific, 10131035). Cell suspensions were centrifuged at 3500×*g* for 40 min. Cell supernatant was transferred to new tubes, and 0.5–1 mL dry volume of Protein A beads (Thermo Fisher Scientific, 20333) per culture was added, followed by overnight gentle rocking at 4 °C. Protein A beads were separated from media using vacuum filter columns (Thermo Fisher Scientific, 89898). The beads were then washed with 75–150 mL of PBS. Antibodies were eluted from Protein A beads using 0.1 M glycine buffer (pH 3.0) and then neutralized with 1 M Tris to a final pH of 7.4. Antibodies were then filtered using 0.2 µm filters, aliquoted and stored at −80 °C. Antibody absorbance at 280 nm was measured and antibody size was evaluated by SDS-PAGE (Thermo Fisher Scientific, WG1203BOX). SDS-PAGE gel images were acquired using a lightbox (Kaiser Slimlite Plano 5000 K 8 × 11”) and camera (iPhone 11).

### Affinity analysis

For affinity analysis, 0.3 µg of biotinylated RBD was immobilized on 3 × 10^7^ streptavidin Dynabeads in PBSB (PBS with 1 g/L BSA) in a final volume of 1.2 mL. Protein and beads were incubated at room temperature for 2–3 days and then stored at 4 °C. For the binding study, beads were washed twice with PBSB and blocked with 10% milk in PBSB by end-over-end mixing at room temperature for 1 h followed by another wash with PBSB. 10^5^ beads/well were incubated with varying concentrations of antibodies in 1% milk in PBSB at room temperature for 2–3 h. Post incubation, the beads were washed once by centrifugation, followed by incubation with goat anti-human IgG AF647 (Jackson ImmunoResearch, 109-605-098) on ice for 4 min. After labeling, beads were washed once with ice-cold PBSB and evaluated by flow cytometry.

### SARS-CoV-2 pseudovirus neutralization assay

The pseudovirus preparation and SARS-CoV-2 neutralization assay were modified from a previous protocol^22^. To prepare virus particles, Lenti-X 293T cells (Takara, 632180) were seeded at 6 × 10^5^ per well in 6-well plates in RPMI media containing supplements of 10% Fetal Bovine Serum (FBS) and 1% penicillin/streptomycin (P/S). The cells were cultured at 37 °C with 5% CO_2_ until reaching a target confluency of 50–70%. Cells were then transfected using lipofectamine 2000 and third generation lentivirus plasmids: HDM-Hgpm2 plasmid (BEI number NR-52517) encoding HIV Gag-Pol under CMV promoter (0.22 µg), HDM-tat1b plasmid (BEI, NR-52518) encoding HIV Tat under CMV promoter (0.22 µg), pRC-CMV-Rev1b plasmid (BEI number NR-52519) encoding HIV Rev (0.22 µg), pHAGE-CMV-Luc2-IRES-ZsGreen-W (BEI number NR-52516) lentiviral transfer plasmid encoding co-expression of luciferase and ZsGreen (1.00 µg), pCMV3 SARS-CoV2 S Untagged Delta 19AA C-term plasmid encoding the SARS-CoV-2 spike (S) protein with a 19-amino acid deletion at the C-terminus (0.34 µg). Cell media was exchanged to fresh RPMI with 10% FBS and 1% P/S at 24 h post-transfection. Then, to isolate and concentrate SARS-CoV-2 pseudovirus (without ultracentriguation), cell supernatant was collected and pressed through a 0.45 µm filter at 72 h post-transfection. Lenti-X Concentrator (Takara, 631232) was added to supernatant at a volume ratio of 1:3 and incubated at 4 °C overnight. To concentrate pseudovirus, the mixture was centrifuged at 1500×*g* for 45 min. Supernatant was removed, and the virus pellet was resuspended in a volume of 50 µL of Opti-MEM per well of virus harvest.

To determine the quantity of Tissue Culture Infectious Units (TCIU) per mL of virus, 293T-ACE2 cells (BEI, NR-52511) were seeded at 10,000 cells per well in a 96-well plate in DMEM with 10% FBS and 1% P/S, at 37 °C and 5% CO_2_. 24 h after seeding, the cells were infected with various dilutions of virus, diluted in DMEM media in the presence of 5 µg/mL polybrene, 10% FBS, and 1% P/S. Representative cell counts per well were also determined at 24 h post seeding. Then, the percentage of ZsGreen-expressing cells was determined via flow cytometry using a Bio-Rad ZE5 cell analyzer and was further verified using fluorescence microscopy at 48 h post-infection.

For the pseudovirus neutralization assays, 293T-ACE2 cells were seeded at 10,000 cells per well in white bottom 96-well plates (Corning, 3917) in DMEM (10% FBS and 1% P/S) and cultured at 37 °C and 5% CO_2_. At 24 h post-seeding, 293T-ACE2 cells were treated with a final concentration of 5 µg/mL polybrene, and mixtures containing 350 TCIU SARS-CoV-2 pseudovirus per well and antibody treatments (fourfold serial dilutions). The mixtures of antibody and SARS-CoV-2 pseudovirus were incubated together for 1 h at 37 °C prior to addition to 293T-ACE2 cells. Next, neutralizing activity was determined via bioluminescence detection using a microplate reader at 48 h post-infection. For this procedure, luciferase substrate (Promega ONE-Glo, E6110) was used following the manufacturer’s protocol. Specifically, 96-well plates were equilibrated to room temperature for 10 min and the media volume in each well was reduced to 100 µL. Luciferase substrate was prepared and added (100 µL per well). The plates were incubated at room temperature for 10 min, and bioluminescence was measured (500 ms integration/well) using a Molecular Devices SpectraMax microplate reader.

### Specificity analysis

To evaluate the affinity of antibodies, His-tag labeled RBD of SARS-CoV and SARS-CoV-2 virus were separately immobilized on microbeads (Thermo Fisher, 10103D). 96-well plates containing RBD-coated beads were incubated with either 13I1 antibodies or VHH-72 nanobodies over a range of concentrations (3 pM to 12.5 nM) in PBSB for 2 h at room temperature. After primary incubation, the beads were washed with ice-cold PBSB and incubated with goat anti-human Fc AF 488 (Jackson ImmunoResearch, 109-545-008) for 4 min on ice. Following secondary incubation, the beads were washed twice with ice-cold PBSB and analyzed by flow cytometry. Similarly, 13I1 coated beads were assessed for binding to WT SARS-CoV-2 S1 protein as well as the S1 protein of variants of concern (B.1.1.7 and B.1.351) using commercially-available antigens (Acro Biosystems; SPD-C52H3, SPD-C52Hn, SPD-C52Hp). Briefly, 13I1-coated microbeads were blocked in PBSB with 10% milk for 1 h, washed with PBSB, and incubated with SARS-CoV-2 S1 proteins in 96-well plate format for 3 h at 25 °C and 225 rpm. Plates were washed with PBSB, incubated with His-tag antibody (Invitrogen, PA-9531) for 30 min on ice, washed with PBSB, incubated with detection antibody (Jackson ImmunoResearch, 703-606-155) for 4 min on ice, washed with PBSB, and analyzed by flow cytometry.

### Competitive binding analysis

To evaluate the epitope of 13I1, competitive binding analysis was performed with ACE2 receptor and other published SARS-CoV-2 antibodies. Biotinylated RBD (5 nM) was first incubated with soluble antibodies or ACE2 over a range of concentrations (0.05, 0.5, 5, 50 and 500 nM) at room temperature for 2 h. Next, the antibody-RBD complexes were incubated with 13I1-coated microbeads in PBSB with 1% milk at room temperature for 3 h. After incubation, beads were washed with cold PBSB followed by incubation with streptavidin AF647 (1:1000) for 4 min on ice. After secondary incubation, the beads were washed twice with cold PBSB and analyzed by flow cytometry.

### Melting temperature analysis

Antibody melting temperatures were measured using differential scanning fluorimetry. Briefly, antibodies were prepared at 0.12 mg/mL in PBS and combined with Protein Thermal Shift Dye (Applied Biosystems, 4461146) at a volume ratio of 7:1 antibody:dye. Background samples were prepared by mixing 1 × PBS with dye at the same ratio. The average of 2–3 PBS-dye mixtures was used to calculate background signal. The antibody-dye and PBS-dye mixtures were added to clear 384-well plates. Plates were submitted to the University of Michigan Advanced Genomics core for analysis. The 384-well plate was centrifuged at 1000–2000 rpm for 1 min and inserted into an ABI Prism 7900HT Sequence Detection System (Applied Biosystems). Thermal cycle conditions analyzed increasing temperatures between 25 and 98 °C over 45 min. Background signals were subtracted from sample signals during analysis. Melting temperatures were determined from the temperatures at which the maximum signals were observed (first derivatives equal to zero). In the case of the DVD construct, the first local maximum value was used to determine the melting temperature when two transitions were observed.

### Analytical size-exclusion chromatography

Antibody purity after Protein A purification was analyzed using size-exclusion chromatography with a Shimadzu Prominence HPLC System outfitted with a LC-20AT pump, SIL-20AC autosampler and FRC-10A fraction collector. Antibodies in 20 mM acetate (pH 5) were buffer exchanged into PBS (pH 7.4). For analytical SEC, 100 µL of sample (diluted to 0.1 mg/mL) was loaded onto the column (Superdex 200 Increase 10/300 GL column; GE, 28990944) and evaluated at a flow rate of 0.75 mL/min using a PBS running buffer supplemented with 200 mM arginine (pH 7.4). Absorbance at 280 nm signal was monitored and used for analysis. The percentage of protein monomer was evaluated by analyzing the area under the peak between the exclusion volume and solvent elution times (8 to 22 min).

### Polyspecificity analysis

Polyspecificity reagent (PSR) was prepared as previously reported^27^. Briefly, CHO cells (10^9^, Gibco, A29133) were pelleted, washed with PBSB, washed again with Buffer B (50 mM HEPES, 0.15 M NaCl, 2 mM CaCl_2_, 5 mM KCl, 5 mM MgCl_2_, 10% Glycerol, pH 7.2), and then pelleted. The cell pellets were resuspended in 5 mL of Buffer B with supplementary protease inhibitor (Sigma Aldrich, 4693159001). The cells were homogenized for 90 s (three 30 s cycles) and then sonicated for 90 s (three 30 s cycles). The cell suspension was then centrifuged at 40,000×*g* for 1 h. The supernatant was then removed and discarded.

The pellet (enriched cell membrane fraction) was suspended in Buffer B with a Dounce homogenizer for 30 strokes. Protein concentration was determined using a detergent compatible protein assay kit (BioRad, 5000116). The enriched membrane fraction was diluted to a concentration of 1 mg/mL in solubilization buffer (pH 7.2) containing 50 mM HEPES, 0.15 M NaCl, 2 mM CaCl_2_, 5 mM KCl, 5 mM MgCl_2_, 1% n-dodecyl-b-d-maltopyranoside (Sigma Aldrich, D4641), and a protease inhibitor (Sigma Aldrich, 11873580001). The solution was then mixed overnight at 4 °C via end-over-end mixing. The soluble membrane protein fraction was then centrifuged at 40,000 xg for 1 h and the supernatant was collected. The final concentration of supernatant was measured again and diluted to 1.0 mg/mL.

Sulfo-NHS-LC-biotin (Thermo Fisher Scientific, PI21335) was dissolved in distilled water at ~ 11.5 mg/mL. Stock solutions of Sulfo-NHS-LC-biotin (150 mL) and PSR reagent (4.5 mL at 1.0 mg/mL) were mixed end-over-end at room temperature for 45 min. To quench the reaction, 10 mL of 1.5 M hydroxylamine at pH 7.2 was added. Biotinylated PSR was then aliquoted and stored at −80 °C.

Protein A-coated magnetic beads (Invitrogen, 88846) were washed three times with PBSB and then incubated with antibodies or nanobodies at various concentrations ranging from 0.03 × to 10 × of the saturated bead binding capacity for IgGs in 96-well plates (VWR, 650261) overnight at 4 °C. Antibody and nanobody concentrations were normalized by molarity to maintain the same Fc concentration across the samples. The IgG-coated beads were washed twice with PBSB, with centriguation at 2500×*g* for 4 min between washing steps. Next, the beads were suspended with a 10 × diluted solution of biotinylated PSR and incubated for 20 min on ice. Following this incubation, beads were washed once with PBSB and then incubated with a 1000 × dilution of streptavidin AF-647 (Invitrogen, S32357) and a 1000 × dilution of goat anti-human Fc[F(ab’)_2_] AF-488 (Invitrogen, H10120) for 4 min on ice. Beads were washed once, resuspended in PBSB, and evaluated via flow cytometry. The high and low non-specific binding control antibodies used in this assay have the variable regions of emibetuzumab and elotuzumab grafted onto a common IgG1 framework, respectively. The control antibodies were two-step purified by Protein A and SEC. Results from all replicates were normalized between 0 and 1 based on control antibodies.

## Supplementary Information

Supplementary Information.

## Data Availability

All data generated or analyzed during this study are included in this published article (and its Supplementary Information files).
