# Supplementary material for: Discovery and characterization of high-affinity, potent SARS-CoV-2 neutralizing antibodies via single B cell screening
Source: Sci Rep. 2021 Oct 20;11:20738. doi: 10.1038/s41598-021-99401-x (PMC8528929; doi:10.1038/s41598-021-99401-x)
Supplement: Supplementary file 1 — Supplementary Information. [file 41598_2021_99401_MOESM1_ESM.pdf]

Supplementary Information for:

Discovery and characterization of high-affinity, potent SARS-CoV-2 neutralizing antibodies via single B cell screening

John S. Schardt[1,2,4,†], Ghasidit Pornnoppadol[2,4,†], Alec A. Desai[1,4], Kyung Soo Park[3,4], Jennifer M. Zupancic[1,4], Emily K. Makowski[2,4], Matthew D. Smith[1,4], Hongwei Chen[1,2,4], Mayara Garcia de Mattos Barbosa[5], Marilia Cascalho[5,6], Thomas M. Lanigan[7], James J. Moon[2,3,4], Peter M. Tessier[1,2,3,4*]

[1]Departments of Chemical Engineering, [2]Pharmaceutical Sciences, and [3]Biomedical Engineering,
[4]Biointerfaces Institute, University of Michigan, Ann Arbor, MI 48109, USA
[5]Department of Surgery, University of Michigan, Ann Arbor, MI, 48109, USA
[6]Department of Microbiology and Immunology, University of Michigan, Ann Arbor, MI, 48109, USA
[7]Division of Rheumatology, Department of Internal Medicine, University of Michigan Medical School, Ann Arbor, Michigan, USA

*To whom correspondence should be addressed: Peter M. Tessier

Address: North Campus Research Complex, B10-179
2800 Plymouth Road
University of Michigan
Ann Arbor, MI 48109
Email: ptessier@umich.edu
Phone: +1 (734) 763-1486

†Co-first author

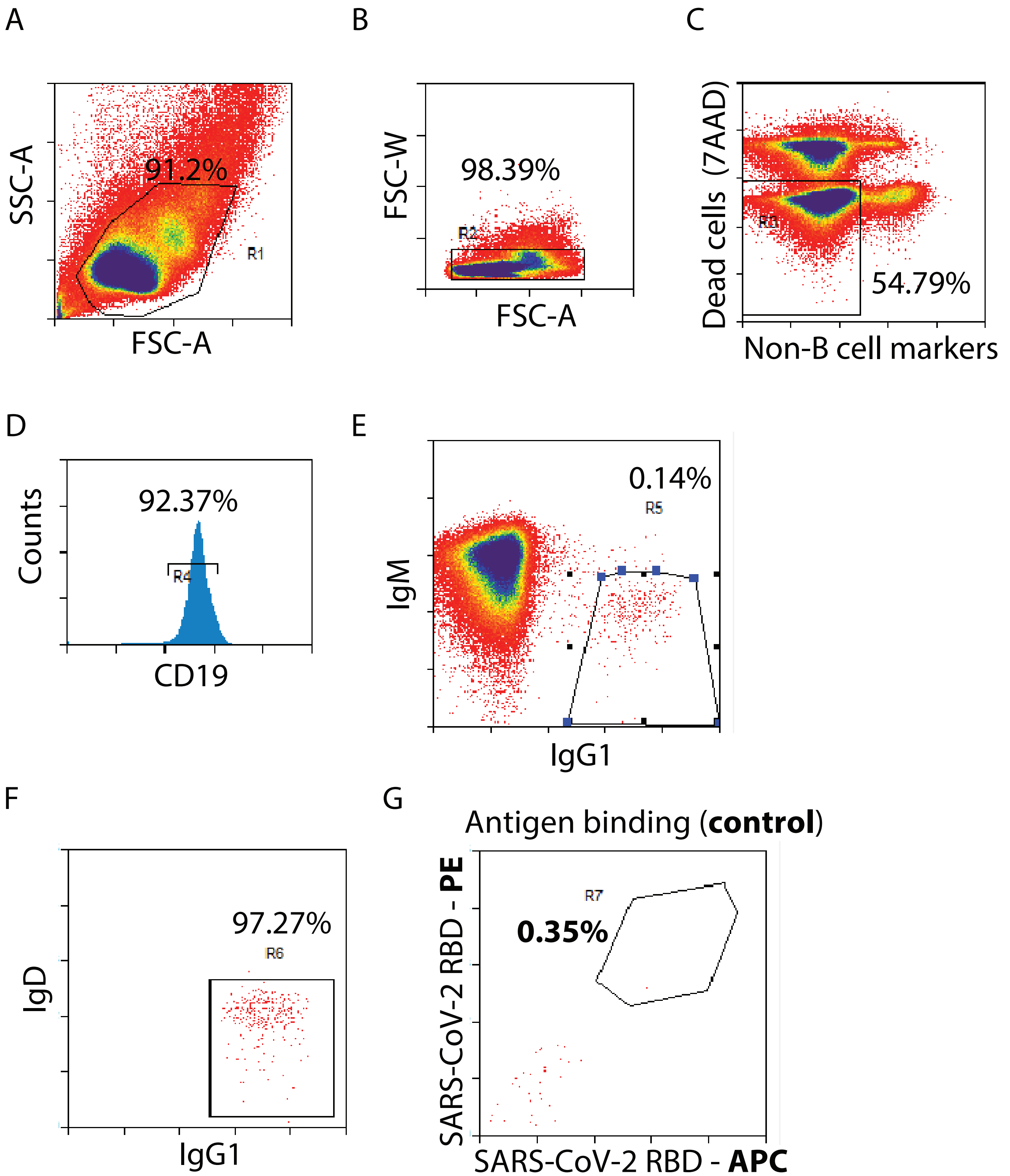

Supplementary Figure 1. Flow cytograms for a naïve control mice sample. Flow cytograms of cell samples from **(A-G)** control naïve mice are shown. The following **(A-G)** gating strategy was applied in series: **(A)** selection of lymphocytes based on size (forward scatter area) and granularity (side scatter area), **(B)** doublet discrimination based on forward scatter width and forward scatter area, **(C)** viability and lack of non-B cell markers (T cell marker CD4, T cell marker CD8, neutrophil marker GR-1, macrophage marker F4/80), **(D)** B cell marker CD19, **(E)** negative for IgM (naïve B cell marker) and positive for IgG1, **(F)** negative for IgD (naïve B cell marker), **(G)** binding to SARS-CoV-2 RBD labeled with PE and APC fluorescent proteins.

A

```
12H2      EVQLQESGPGLVKPSQSLFLTCTVTGYSITSDYAWNWIRQFPGNKLEWMGYITYS-GSTS    59
13I1      EVQLQESGPGLVKPSQSLSLTCTVTGYSITSDYAWNWIRQFPGNKLEWMGYITYS-GSTS    59
4A7       EVQLQESGGGLVQPGGSLKLSCAASGFDF-SRCWMSWVRQAPGKGLEWIGEINPDSSTIN    59
1A1       EVQLQESGGGLVRPGGSLKLSCAASGFIF-SRYAMSWVRQTPEKRLEWVASIRSG-GDTY    58
6C5       EVQLQESGGGLLQPGRSLKLSCVASGISF-SNYDMAWVRQSPTKGLEWVASISTGGGNTD    59
          ******** **::*. ** *:*..:*  : *     *:** * : ***:. *  . .

12H2      YNPSLKSRISITRDTSKNQFFLQLNSVTTEDTATYYCARGDGYF--FPLDYWGQGTTLTV    117
13I1      YNPSLRSRISITRDTSKNQFFLQLNSVTSEDTATYYCARGDGYY--FPLDYWGQGTTLTV    117
4A7       YTDSLKDKFIISRDNAKNTLYLQMSKVRSEDTALYYCARPE--YYGNYFDYWGQGTTLTV    117
1A1       YPDSVKGRFTISRDDARNILYLQMNSLRSEDTAMYYCAS----FITSAKDYWGQGTTLTV    114
6C5       YRDSVKGRFTISRDNAKSTLYLQMDSLRSEDTATYYCSRGGYGYTSRGFTYWGQGTLVTV    119
          *  *::.:: *:** ::. ::**:..: :**** ***:     :      ****** :**

12H2      SS   119
13I1      SS   119
4A7       SS   119
1A1       SS   116
6C5       SS   121
          **
```

B

```
12H2      DIVMTQSPSSLSVSAGEKVTMSCKSSQSLLNSGNQKSYLAWYQQRPGQPPKLLIYGASTR    60
13I1      DIVMTQSPSSLSVSAGEKVTMSCKSSQSLLNGGNQKSYLAWYQQKPGQPPKLLIYGASTR    60
4A7       DIVMSQSPSSLAVSAGEKVTMSCKSSQSLLNSRTRKNYLAWYQQKPGQSPKLLIYWASTR    60
1A1       DIVMSQSPSSLAVSAGEKVTMSCKSSQSLLNSRTRKNYLAWYQQKPGQSPKLLIYWASTR    60
6C5       DIVMSQSPSSLAVSAGEKVTMSCKSSQSLLNSRTRKNYLAWYQQKPGQSPKLLIYWASTR    60
          ****:******:*******************. .:*.*******:*** ****** ****

12H2      ESGVPDRFTGSGSGTDFTLTISSVQAEDLAVYYCQNDHSYPPTFGAGTKLELK  113
13I1      ESGVPDRFTGSGSGTDFTLTISSVQAEDLAVYYCQNDHSYPPTFGAGTKLELK  113
4A7       ESGVPDRFTGSGSGTDFTLTISSVQAEDLAVYYCKQSYN-LLTFGAGTKLELK  112
1A1       ESGVPDRFTGSGSGTDFTLTISSVQAEDLAVYYCKQSYN-LLTFGAGTKLELK  112
6C5       ESGVPDRFTGSGSGTDFTLTISSVQAEDLAVYYCKQSYN-LLTFGAGTKLELK  112
          **********************************::.:.   ***********
```

Supplementary Figure 2. Multiple amino acid sequence alignments of V_H and V_L genes of selected antibodies. A. Multiple sequence alignment of **(A)** V_H genes and **(B)** V_L genes**.** Alignments generated using Clustal Omega Multiple Sequence Alignment tool. "*" indicates perfect alignment; ":" indicates similar residues; "." indicates weakly similar residues.

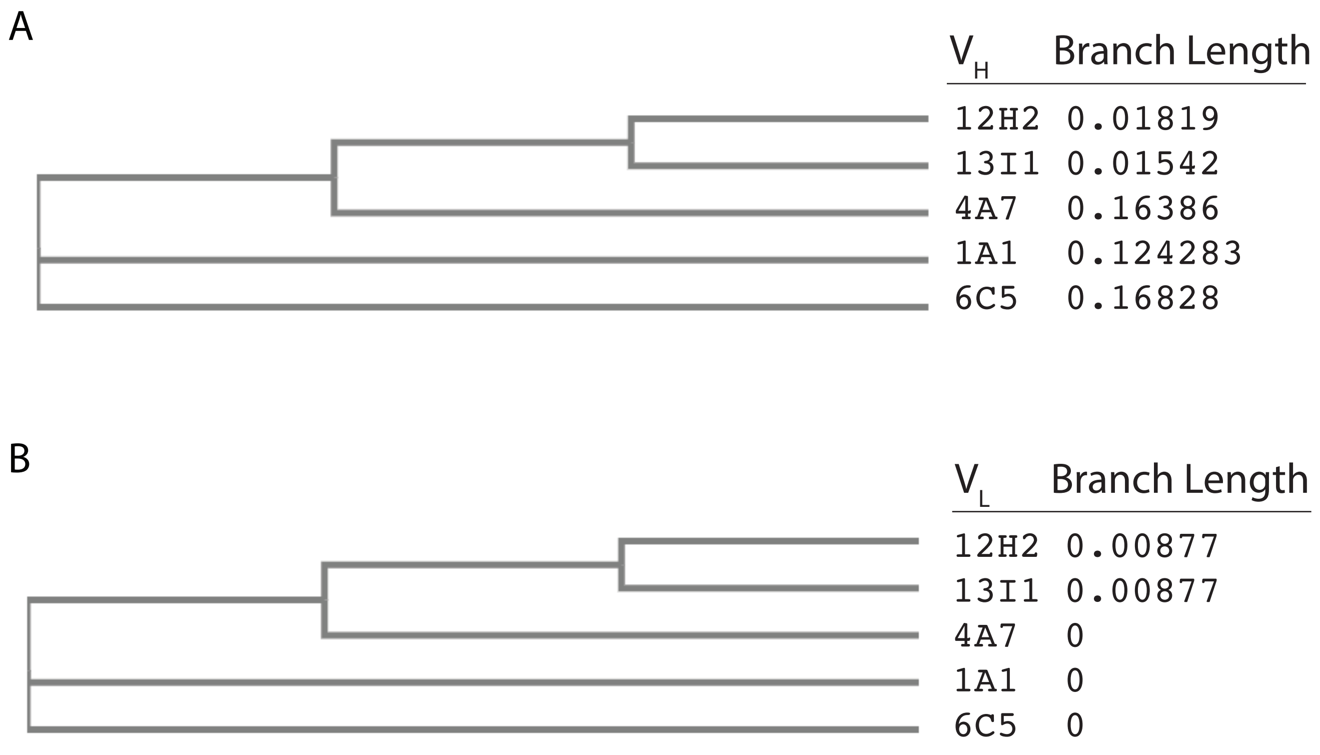

Supplementary Figure 3. Phylogenetic amino acid sequence analysis of isolated antibodies. Phylogenetic neighbor-joining trees without distance corrections are depicted for **(A)** V_H and **(B)** V_L amino acid sequences using Clustal Omega Multiple Sequence Alignment Tool. Branch length values, indicating evolutionary distance between sequences, are shown for each antibody variable region. A branch length of zero indicates an identical sequence.

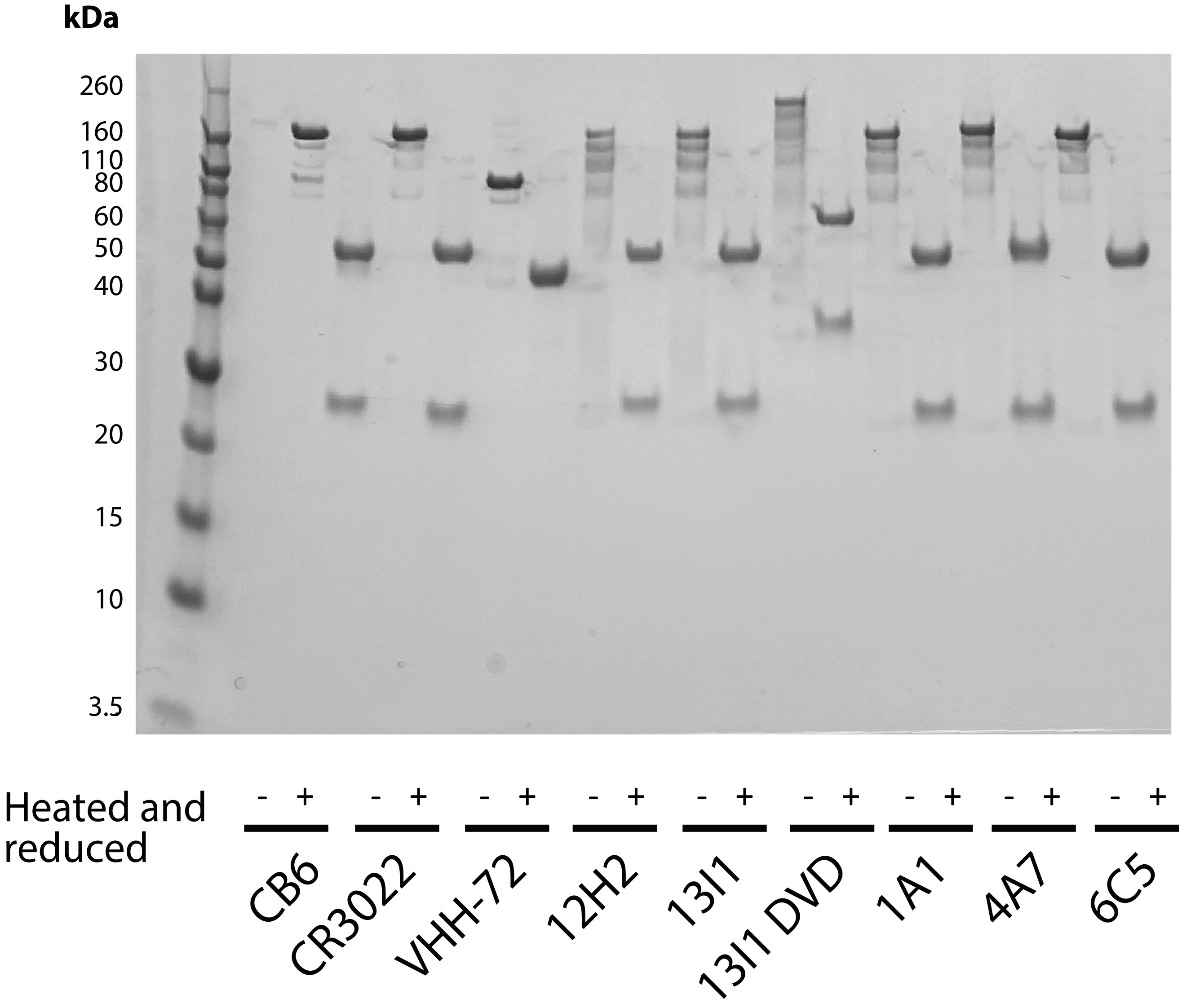

Supplementary Figure 4. SDS-PAGE analysis of the panel of antibodies evaluated in this work. Prior to sample loading, antibodies were prepared under untreated (-) or heated and reduced (+) conditions. Samples were loaded at 3 μg per well and stained with Coomassie reagent. The SDS-PAGE gel image was acquired using a lightbox (Kaiser Slimlite Plano 5000K 8x11") and camera (iPhone 11). The gel image was cropped.

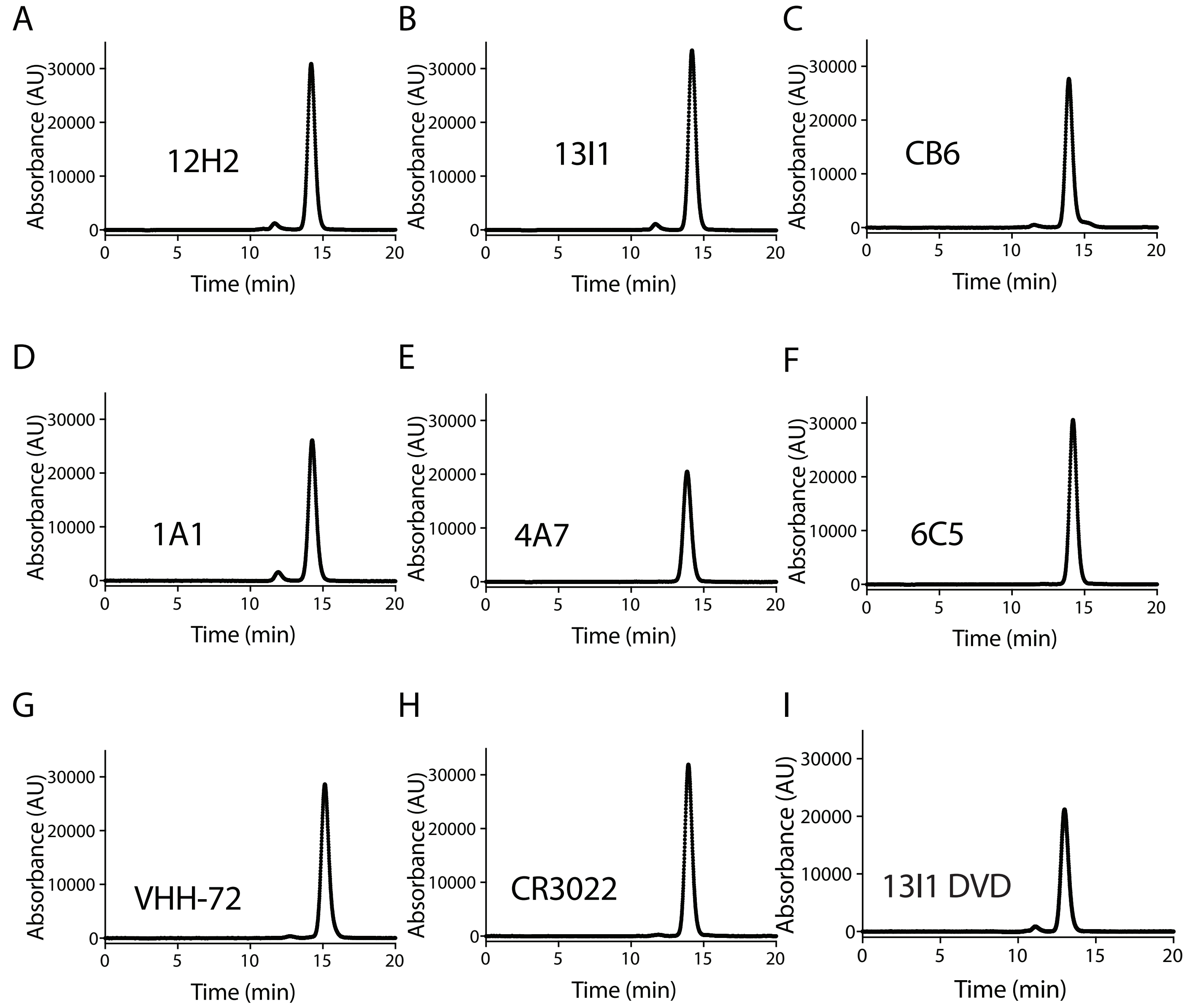

Supplementary Figure 5. Analytical size exclusion chromatography reveals high purity and confirms predicted molecular weights for the antibody panel used in this study. SEC profiles are presented as follows: **A.** 12H2 **B.** 13I1 **C.** CB6 **D.** 1A1 **E.** 4A7 **F.** 6C5 **G.** VHH-72 **H.** CR3022. **I.** 13I1 DVD. The running buffer was PBS (pH 7.4) with 200 mM arginine.

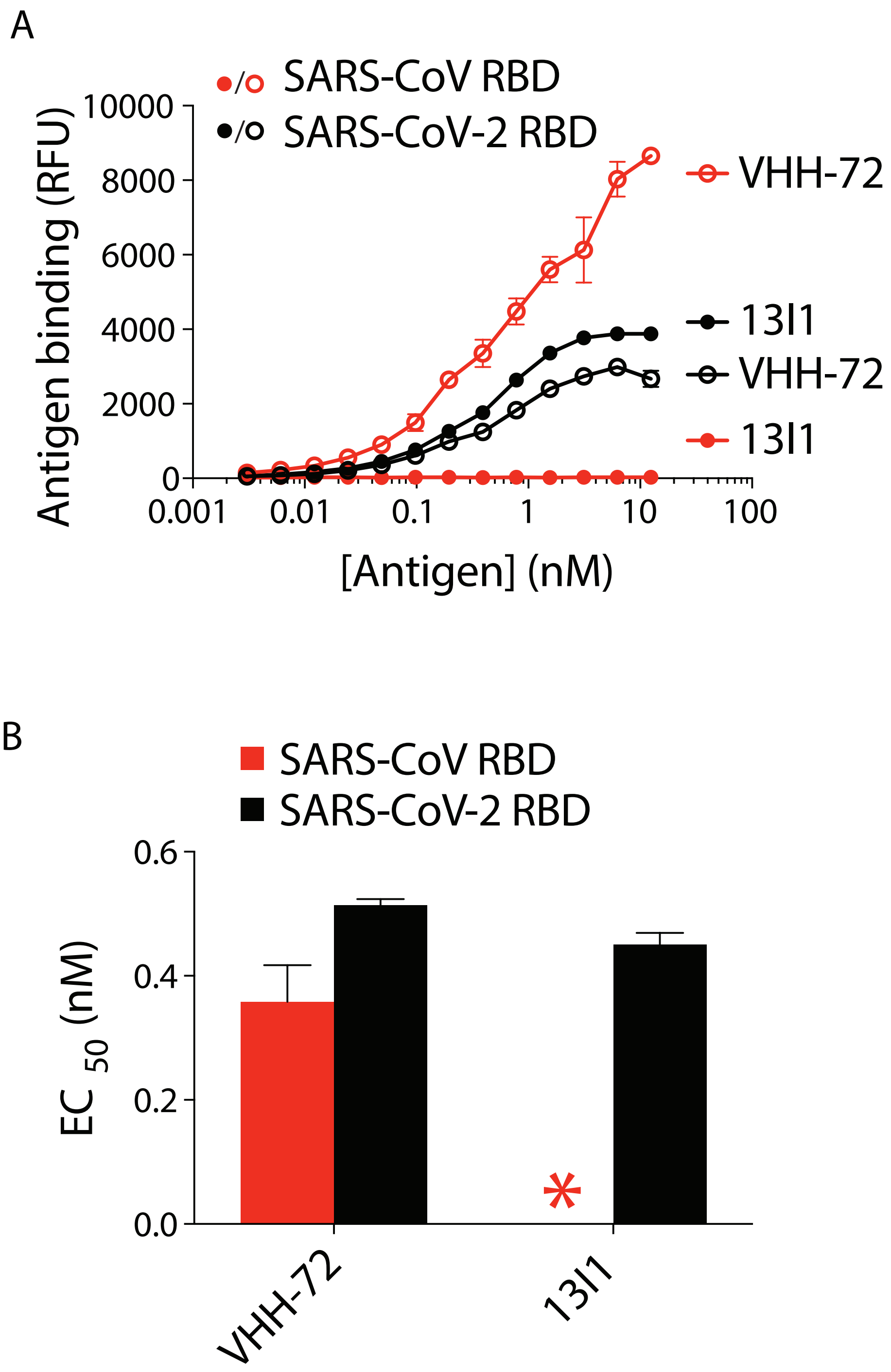

Supplementary Figure 6. Neutralizing antibody 13I1 selectively recognizes SARS-CoV-2 RBD. A. Binding response to the RBD of SARS-CoV and SARS-CoV-2 was evaluated. Antibodies were immobilized on Protein A beads and binding to the RBDs of SARS-CoV and SARS-CoV-2 was evaluated via flow cytometry. VHH-72 (as an Fc fusion protein) was also evaluated as a positive control because it recognizes both SARS-CoV and SARS-CoV-2 RBDs. **B.** Half-maximal effective concentration (EC_{50}) values for antibody binding to the RBD of SARS-CoV and SARS-CoV-2. The results are averages from two independent experiments and the error bars are standard deviations.

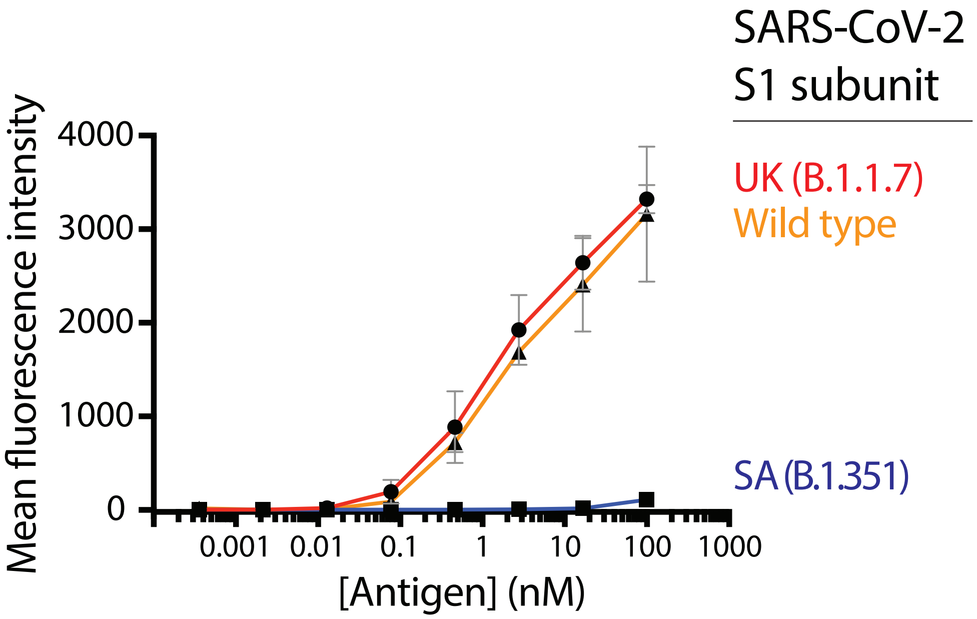

Supplementary Figure 7. Affinity analysis of 13I1 antibody binding to variants of the SARS-CoV-2 S1 subunit. 131 antibody immobilized on microbeads was assessed for binding to soluble SARS-CoV-2 S1 subunit protein of the wild type (Wuhan strain), United Kingdom (UK) variant B.1.1.7 and South African variant (SA) B.1.351 by flow cytometry. Data are mean of two independent experiments. Error bars are standard deviations.

Table S1. Summary of antibodies used for fluorescence-activated cell sorting. Antibody target, cell type, and fluorophore conjugate are listed below. Table S1 is adapted from a previous report.[1]

Antibody target	Cell type	Fluorophore
CD19	B cells	AF700
CD8	T cells	FITC
CD4	T cells	FITC
GR1	Neutrophils	FITC
F4/80	Macrophages	FITC
IgG1	Memory B cells	BV421
IgM	Naïve B cells	PE/Cy7
IgD	Naïve B cells	AP/Cy7

Table S2. Composition of reverse transcription mixtures I and II.

Mixture I

Component	Volume (μL)	Final concentration
Nuclease-free water	661	
IGEPAL (10%)	47	
Random primers (300 ng/μL)	47	18.3 ng/μL
RNasin + RNase inhibitor (40 U/ μL)	15	0.8 U/ μL
Total	770	

Mixture II

Component	Volume (μL)	Final concentration
Nuclease-free water	236.5	
5x First-strand buffer	330	2.3x
DL-DTT (100 mM)	110	14.3 mM
dNTP (25 mM)	55	1.8 mM
RNasin + RNase inhibitor (40 U/μL)	11	0.6 U/μL
SuperScript III RT (200 U/μL)	27.5	7.1 U/μL
Total	770	

Table S3. Thermocycler incubation program for cDNA synthesis.

Temperature (°C)	Duration (min)
42	10
25	10
50	60
94	5
4	∞

Table S4. First PCR amplification of V_H and V_L genes.

Components for first PCR amplification for one 96-well plate:

Component	Volume (μL)	Final concentration
Nuclease-free water	3,328	
10x Buffer<br>(supplied with HotStar Taq polymerase)	384	1x
dNTP (25 mM)	48	0.3 mM
5' Forward primers	23	0.3 μM
3' Reverse primers	15	0.2 μM
HotStar Taq polymerase (250 U/ 50 μL)	42	0.05 U/μL
Total	3,840	

Thermocycler program for first PCR amplification

Number of cycles	Denature (94°C)	Anneal (46°C)	Extend (72°C)
1	5 min		
50	30 s	30 s	55 s
1			10 min

Table S5. Second PCR amplification (Seq-PCR) of V_H and V_L genes.

Components for Seq-PCR for one 96-well plate:

Component	Volume (μL)	Final concentration
Nuclease-free water	2,536	
Loading buffer	800	
10x Buffer (supplied with HotStar Taq polymerase)	384	1x
dNTP (25 mM)	48	0.3 mM
5' Forward primers (50 μM)	15	0.2 μM
3' Reverse primers (50 μM)	15	0.2 μM
HotStar Taq polymerase (250 U/ 50 μL)	42	0.05 U/μL
Total	3,840	

Thermocycler program for Seq-PCR amplification

Number of cycles	Denature (94°C)	Anneal (57°C)	Extend (72°C)
1	5 min		
50	30 s	30 s	50 s
1			10 min

Tables S2-5 are adapted from previously published literature.[2]

Supplementary Information References

1. Clargo, A. M. *et al.* The rapid generation of recombinant functional monoclonal antibodies from individual, antigen-specific bone marrow-derived plasma cells isolated using a novel fluorescence-based method. *mAbs* **6**, 143–159 (2014).

2. von Boehmer, L. *et al.* Sequencing and cloning of antigen-specific antibodies from mouse memory B cells. *Nat. Protoc.* **11**, 1908–1923 (2016).
